# An Update on Mesoporous Silica Nanoparticle Applications in Nanomedicine

**DOI:** 10.3390/pharmaceutics13071067

**Published:** 2021-07-12

**Authors:** Elham Rastegari, Yu-Jer Hsiao, Wei-Yi Lai, Yun-Hsien Lai, Tien-Chun Yang, Shih-Jen Chen, Pin-I Huang, Shih-Hwa Chiou, Chung-Yuan Mou, Yueh Chien

**Affiliations:** 1 Department of Medical Research, Taipei Veterans General Hospital, Taipei 11217, Taiwan; elham.rastegar@gmail.com (E.R.); yj1007hsiao@yahoo.com (Y.-J.H.); jefflai8228@gmail.com (W.-Y.L.); tony1233000@yahoo.com.tw (Y.-H.L.); yangtienchun@gmail.com (T.-C.Y.); sjchen96@gmail.com (S.-J.C.); 2 Institute of Pharmacology, National Yang-Ming Chiao Tung University, Taipei 11217, Taiwan; 3 School of Medicine, National Yang-Ming Chiao Tung University, Taipei 11217, Taiwan; 4 Department of Ophthalmology, Taipei Veterans General Hospital, Taipei 11217, Taiwan; 5 Department of Oncology, Taipei Veterans General Hospital, Taipei Veterans General Hospital, Taipei 11217, Taiwan; pihuang@vghtpe.gov.tw; 6 Department of Chemistry, National Taiwan University, Taipei 10617, Taiwan

**Keywords:** mesoporous silica nanoparticles, cancer therapy, bioimaging, tissue engineering, stem cell research

## Abstract

The efficient and safe delivery of therapeutic drugs, proteins, and nucleic acids are essential for meaningful therapeutic benefits. The field of nanomedicine shows promising implications in the development of therapeutics by delivering diagnostic and therapeutic compounds. Nanomedicine development has led to significant advances in the design and engineering of nanocarrier systems with supra-molecular structures. Smart mesoporous silica nanoparticles (MSNs), with excellent biocompatibility, tunable physicochemical properties, and site-specific functionalization, offer efficient and high loading capacity as well as robust and targeted delivery of a variety of payloads in a controlled fashion. Such unique nanocarriers should have great potential for challenging biomedical applications, such as tissue engineering, bioimaging techniques, stem cell research, and cancer therapies. However, in vivo applications of these nanocarriers should be further validated before clinical translation. To this end, this review begins with a brief introduction of MSNs properties, targeted drug delivery, and controlled release with a particular emphasis on their most recent diagnostic and therapeutic applications.

## 1. Introduction

Mesoporous Silica Nanoparticles (MSNs) are porous solid materials with inorganic siloxane structures that have garnered increasing attention as an ideal candidate for therapeutic applications [1,2,3]. MSNs are known as nanocarriers with tunable pore diameters in the range of 2–50 nm [4,5,6]. These nanocarriers possess remarkable physicochemical properties, including large surface area (>600 m^2^/g) and pore volume (>0.6 cm^3^/g) [7]. In addition, low mass density, controllable nanoparticle size (50–200 nm)/shape, easy synthesis, and large-scale (0.5 kg) production make them promising therapeutic and diagnostic candidates [8,9]. These favorable characteristics allow researchers to selectively load multiple cargos with different sizes and unload them with the desired release on demand. Notably, the internal and external surfaces of MSNs make them compatible with many different types of modifications and functionalization in a selective fashion [10,11]. For example, the modification of MSNs by conjugating luminescent agents or incorporating magnetic nanoparticles offers the possibility of multi-delivery of the drugs and imaging agents [12,13]. This approach allows the practical tracking of drug delivery and thereby improving the therapeutic efficacy [14]. In addition, the superior biocompatibility, biodegradability, and clearance of these nanocarriers provide an essential basis for multifaceted therapeutic applications [15,16,17]. It is worth mentioning that MSNs are resistant to a wide range of stresses, including pH, mechanical, and thermal stresses [18,19]. More importantly, MSNs are “Generally Recognized as Safe (GRAS)” by United States Food and Drug Administration (USFDA), which makes them eligible for therapeutics treatments [20,21,22,23]. Therefore, MSNs have been extensively explored for their applications, such as drug delivery, due to their enhanced efficacy and non-toxic effect over the last few decades [24,25]. Besides their conventional drug delivery applications, MSNs have been shown to be promising candidates in several clinical applications, including the diagnosis and treatment [26,27,28,29]. This review briefly introduced MSN synthesis and classifications (Section 2) and the fundamentals of MSN-based drug delivery, including the functionalization, loading, delivery, and release of drugs (Section 3). We further updated and summarized MSN applications in nanomedicines, including the applications in tissue engineering, bioimaging, stem cell research, and anti-cancer/tumor therapy (Section 4; Table 1). Furthermore, we also updated the current consideration regarding MSNs biocompatibility and safety.

## 2. MSN Synthesis and Classification

**Figure 1:**
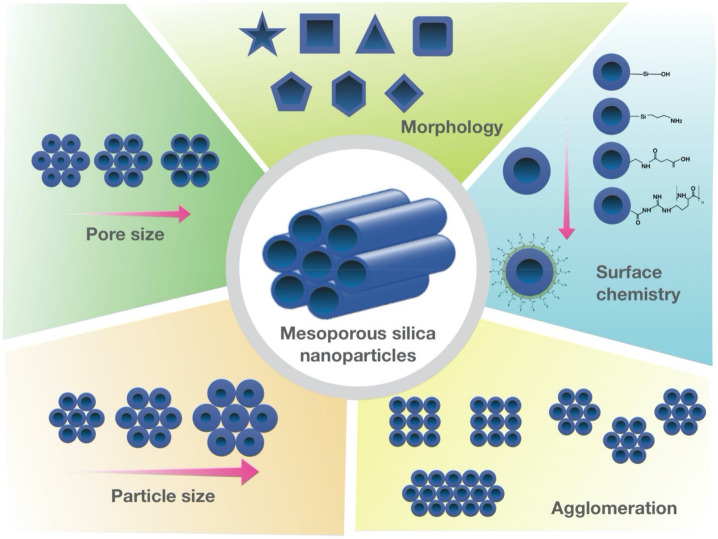
**Tunable physicochemical properties of MSNs.** Schematic illustration of MSNs with various pore sizes and structures, surface chemistry, agglomeration patterns, and different morphologies.

**Figure 2:**
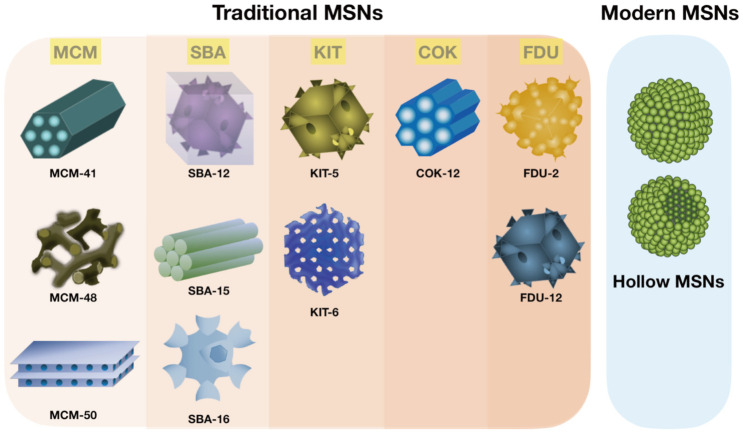
**The construction of MSNs via different strategies.** Schematic classification of material-based traditional MSNs and modern hollow MSNs.

The tetrahedral architecture of silica is formed by covalent bonds between silicon and the surrounding four oxygens in which each oxygen is shared between two silicon atoms [30]. As previously mentioned, due to the flexible bonding between constituent elements, the silica atoms can arrange into a diverse type of orders, which contributes to their growing applications [31,32]. The silica material can be classified into three main categories based on the pore diameter, including microporous (<2 nm), mesoporous (2–50 nm), and macroporous (>50 nm) [33,34,35,36]. In this review, we only focus on mesoporous silica and its therapeutic applications. Since the discovery of MSNs, different synthesis methods (e.g., the Sol–Gel method [37,38,39], the hydrothermal method [40,41,42,43], and the green method [44,45,46,47,48]) have been developed to enhance their efficiency according to specific applications. Here, we briefly introduce the templating method that has been widely used to synthesize different types of MSNs, known as sol-gel synthesis. Surfactants (templates), silica precursors, catalysts, and a polymer are major components involved in the template-directed synthesis of MSNs [39,46,48]. The presence of surfactant (e.g., CTAB) is important to induce micellization within the foam generated by vigorous stirring [49,50]. The surfactant chain plays a key role in directing the mesoporous architecture in which silica seeds are coated on the surface of the template and forms a vesicle or micelles structure [51,52,53]. The presence of various liquid crystal morphologies and mesophases in the surfactant structure leads to excellent flexibility and adaptation of MSNs [54]. The addition of a polymer (e.g., nonionic diblock) provides the mesostructure to the foam [55,56]. The oligomeric silicon precursors (silicon alkoxide) are responsible for the formation of the silica structure outside the micelles, and the catalysts facilitate the hydrolysis and condensation of silica precursors to form a network of siloxane bonds [57,58,59]. Mesoporous nanoparticles are obtained after the elimination of surfactant that leads to the opening of the porosity. The synthesis of MSNs can be optimized by various factors, such as the addition of pore swelling agent, the concentration and length of hydrocarbon chain associated with the surfactant, as well as the source and concentrations of silica [60,61]. In addition, the rates of the silica source hydrolysis and condensation are important considerations to direct architectural characteristics of MSNs [31,57,58,59]. In order to control the sizes, mesostructures (e.g., hexagonal, cubic, and lamellar), and morphologies (e.g., rod, spheres, shuttle, and many complex derivatives) of the mesoporous silicas, the interaction between surfactants and silica precursors is of great importance [62,63]. In addition to the surfactant–silica assembly thermodynamics, researchers can adjust the kinetics of sol–gel chemistry (such as the pH, temperature, and the water content of the reaction mixture) to produce MSNs with desired morphologies and dimensions [64,65]. However, the concern regarding the template-based MSNs is that some drugs are absorbed on the flat surfaces of the MSNs rather than being absorbed in the cavities, causing poor loading efficiency and durability [66]. In recent years, a diverse range of MSNs have been developed using different strategies [67,68]. For example, mesoporous silica structures SBA-15 (Santa Barbara Amorphous 15) synthesized using amphiphilic block copolymers as organic structure-directing agents [69,70]. Another interesting design of MSNs is a construct possessing several mesoporous silica layers with various pore sizes, named as hierarchically porous nanoparticles (NPs) [62] (Figure 1). With all these different types of MSNs, choosing the proper one that best fits our intended application is the most important initial step. For example, MSNs with large pore sizes are used to deliver large biomolecules, such as various types of DNA, siRNA, and proteins [71]. Hollow mesoporous silica nanoparticles (HMSNs) with a large hollow space facilitates the drug and protein delivery of MSNs [72,73] (Table 2; Figure 2).

## 3. The Fundamentals of MSN-Based Drug Delivery

In Section 3, we reviewed the fundamentals of MSN-based drug delivery, including the functionalization, loading, delivery, and release of drugs.

### 3.1. Functionalization

Even though MSNs possess unique characteristics, for some applications, a particular modification is required. The unique architecture of MSNs allows selective functionalization of MSNs that introduces new moieties to the existing surface features [65]. In addition to the features such as shape, size, and porosity, surface functionalization is a critical step for the design of MSNs to serve different roles [27,68]. These characteristics affect the particles’ behavior under biological conditions such as dissolution, drug load, and target delivery [29]. One of the remarkable features of MSNs is the presence of both internal and external surfaces, serving as a platform for various modifications, which ultimately determine its therapeutic performance, such as controlled release and targeted delivery of drugs [91,92,93]. Customizing MSNs by conjugating organic and inorganic compounds is a prerequisite for acquiring multifunctional property and can be achieved through two main approaches: co-condensation and post-synthetic modification [58,59,75,94]. Co-condensation as the first approach relies on the incorporation of functional silica precursors inside the pores during nanoparticle synthesis [58,59]. The co-condensation method has several advantages, including its applicability to a wide range of organoalkoxysilanes, the homogeneous coverage of functional groups, its adaptability to various reaction conditions, and the high payload of functional groups with no adverse effect on the structure of mesoporous silica [26,58,59]. Post-synthetic modification, the second approach of functionalization, involves the attachment of various modifying agents carrying functional groups to the external surface, pore surface, and/or pore entrance on the nanoparticle in a selective manner [58,59]. Generally speaking, prior to any surface functionalization, considering the pore volume and surface area of MSNs is a critical step to determine the involved proportion of the surface in the modification step [75,81]. In some cases, the surface of MSNs is attached to the ligands that can specifically bind to target cancer cells with increased expression of the associated receptors [95,96,97]. Furthermore, the covalent attachment of different imaging agents and targeting ligands to MSNs modify their characteristics and thereby therapeutic applications of these nanocarriers [96,98,99]. For example, a magnetic core, such as iron oxide, can be incorporated into MSNs to enhance magnetic resonance imaging (MRI) and modulate their therapeutic outcomes [14,40]. The functionalization of MSNs with hydrophobic groups is widely used for therapeutic purposes as some drugs with a hydrophobic nature hardly penetrate MSNs due to their hydrophilic surface [100]. Besides, one of the common methods for MSN-based delivery of negatively charged nucleic acids is its functionalization through coating the surface with positively charged polymers, such as polyethyleneimine (PEI). This modification ensures the desirable electrostatic interactions as well as protecting nucleic acids from enzymatic degradation [101,102]. The functionalized MSNs with positive charges are able to penetrate bacteria walls and biofilms with negative charges and, therefore, can be combined with antibiotics to provide more effective treatment [103]. MSNs can be conjugated with various ligands, such as growth factors, aptamers, and vitamins, which leads to sequestering of nanoparticles within membrane-bound endosomes [104,105,106]. This results in increased activity of the drug as well as reducing its toxicity [105]. MSNs have also shown promising efficacy in photodynamic therapy, an alternative to radio- and chemotherapies [107,108]. In this approach, the photosensitizers are incorporated into MSNs, while functionalizing them with specific ligands to target cancer cells [97,105,109].

**Figure 3:**
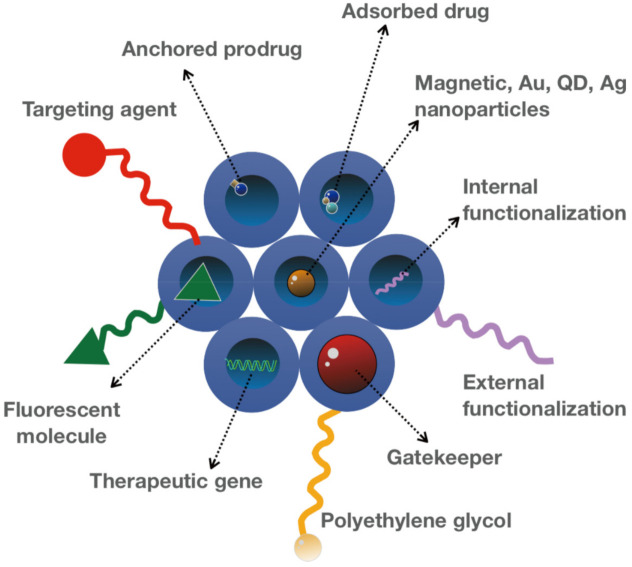
**Functionalization of MSNs.** MSN pores with different surface modifications allow the construction of smart MSNs with controlled drug delivery and release, required for various therapeutic and diagnostic applications.

The functionalization of the peripheral particle surface controls the colloidal and chemical stability as well as specific cell targeting by modifying the moiety of MSNs [1]. The peripheral surface functionalization is crucial for the inclusion of large molecules required for pore gating capacity, which improves the biocompatibility and safety of MSNs [58]. Finally, the surface functionalization allows the co-delivery of cargos with different properties to achieve synergistic effects that can potentiate the therapeutic responses [110]. For example, SOST siRNA and the therapeutic peptide osteostatin were co-delivered by PEI-coated MSNs to treat osteoporosis [101,102,110]. Therefore, the surface of MSNs can be modified in numerous ways to acquire new moieties based on different therapeutic strategies and applications (Figure 3).

### 3.2. Drug Loading

An ideal drug loading involves the absorption of a high concentration of cargo within the internal surface of MSNs and the release at a specific site and timing with minimal waste. With the porous structure, MSNs can encapsulate and shelter cargos that protect them from enzymatic degradation [100,101,102]. Compared with the organic nanocarriers, MSNs have the advantage of a higher loading capacity, mainly due to the enhanced encapsulation efficiency and possibilities for targeted and stimuli-responsive drug delivery [12,111]. However, despite their emerging importance in several biomedical fields, the low clinical success rate is a major concern. Since not every drug can be loaded onto MSNs at a high concentration, a higher dosage of MSNs is required to compensate for this deficiency and achieve the therapeutic effect [112]. This hurdle emphasizes the necessity of the safety evaluation of MSNs prior to the clinical translation regardless of the drug safety [113,114,115,116].

Several methods for loading drugs into MSNs have been developed and reviewed by others [116,117,118]. Physical absorption is the most common method for loading small drugs into MSNs pores, which involves the soaking of MSNs in a drug-containing solution by which the drugs can diffuse into the pore cavities of the particle [119]. The hydrogen bonding and electrostatic interactions between the cargo and the particle are required for the absorption of the cargo onto MSNs. For example, the negatively charged silica surface can serve as the adsorption sites for water-soluble cargos through electrostatic interactions [103]. Surface modifications can further enhance the drug loading capacity by regulating the charge and hydrophilicity of MSNs [100]. As previously mentioned, surface area and pore size can greatly influence the loading capacities of MSNs [61]. For example, spherical- and tubular-shaped MCM-41 are most commonly used for drug delivery as they possess a large surface area and narrow pore diameters [120,121]. The narrow pore diameters allow the selective load of organic compounds onto the particles without premature release [122,123]. Like other nanoparticles, the particle size of MSNs also plays a critical role in cellular uptake and consequently their bioactivity. However, compared to the particle size of MSNs, the pore size seems to be more important for drug loading capacity [124,125]. The repulsive forces between the carrier and cargo represent a major hurdle while loading drugs onto the nanoparticle, which can be managed through the pore size modifications according to the size of the drug [71]. In addition, the pore volume also contributes to the drug loading capacity, as MSNs with larger pore volume can embed a larger quantity of the drug. Since most of the MSNs used in drug delivery possess small sizes, the large DNA cargo is loaded on the surface of nanoparticles [71,101,102]. However, the preparation of MSNs with large pore sizes (20 nm) allows the loading of the DNA within pores [71]. The hierarchical mesoporous and hollow structures of MSNs allow the simultaneous loading of two kinds of drugs with different sizes and water solubilities, suggesting the potential of MSNs for combined targeted therapeutics [126,127]. Compared with traditional MSNs, the HMSNs with higher storage capacity and accessible pore channels offer a higher drug payload capacity and efficiency [128]. Several studies have reported that MSNs could be successfully loaded with both anti-resorptive agents and biopharmaceuticals (e.g., alendronate and osteostatin) for treating osteoporosis and stimulating bone regeneration, respectively [104,129,130,131,132,133]. Overall, the aforementioned preparations for the successful loading of drugs onto MSNs should also be compatible with their survival inside the biological system and the subsequent targeted delivery, which will be discussed in the following section.

### 3.3. Drug Delivery

Successful NP-mediated drug delivery into target organs relies on the prolonged blood circulation time and their stability under physiological conditions, reflecting their efficacy and functionality [134,135]. Similar to other NPs, the injected MSNs in the bloodstream tend to accumulate in the liver and spleen (the macrophage centers), resulting in the poor accumulation of MSNs in the target site [136,137]. In the case of tumor tissues, the enhanced permeation and retention (EPR) phenomenon, the so-called passive targeting, can be employed for the targeted NP delivery into the tumor tissues [124]. In this approach, the injected NPs enter the tumor tissue through its aberrant and poorly formed vasculature, leading to the desirable accumulation of drug-containing NPs in the tumor tissue [135]. MSNs with high drug loading capacity are promising nanocarriers to achieve high drug concentration within the tumor tissue [135]. However, the non-specific absorption of serum proteins onto NPs (opsonization) can cause their rapid elimination from the circulation system [28]. As described before, the size, shape (e.g., rod, disk, and sphere) [62,63], and surface charge of MSNs [138,139] play pivotal roles in determining the fate of NPs in different biological environments affecting both their interaction with cells and systemic biodistribution. Several reports demonstrated that MSNs with a size below 100 nm exhibit optimal levels of internalizations [140,141,142]. Regarding the morphology, there are controversial findings as one study reported that MSNs with spherical morphology show higher cellular uptake than the rod-shaped MSNs, mainly due to the longer time required for wrapping the rod-shaped MSNs with larger surface area [143]. However, another study reported that the higher surface area of rod-shaped MSNs is an advantage for the increased contact area with the cell membrane and hence more favorable for the cellular uptake [114]. Nevertheless, more studies are required to confirm the impact of the shape on MSNs cellular uptake. Interestingly, different lengths of rod-shaped MSNs favor congregations in different tissues. For example, short-rods MSNs tend to retain in the liver, while the spleen is the preferred organ for long-rods MSNs [15,114,144]. In the case of cellular uptake and internalization, long-rod-shaped MSNs display better distribution than the short ones. Furthermore, the surface modifications also affect the cellular uptake mechanism as negatively charged MCM-41 showed a faster endosomal escape than positively charged type MSNs [120]. Beyond the shape and size, the surface area also plays a critical role in targeted delivery, especially in the case of MSNs with large surface area [61]. As mentioned earlier, the non-specific absorption of serum proteins onto the surface of NPs contributes to the opsonization phenomenon, which makes them recognizable by immune cells and eventually leads to their elimination from the bloodstream [145,146]. To address this concern, the modifications of the outer surfaces by certain functional group(s) such as polyethylene glycol (PEG) is a common strategy resulting in stealth NPs that can bypass the instant clearance [147,148].

A more efficient approach for the targeted delivery relies on the biological recognition of a specific molecule on the surface of the target tissue/organ. However, this active approach requires surface-modified NPs as ligands that can interact and bind with overexpressed surface receptors on the target organ [98,149]. This interaction further boosts the EPR effect via specific retention, leading to endocytosis of NPs into target cells shown in most solid tumors [124,150]. The efficiency of this approach tightly depends on the affinity between NPs and organs that is mainly determined by the type and abundance of the conjugated ligands and overexpressed receptors [96,99,151]. Based on the moiety of these receptors, different targeting ligands such as antibodies, peptides, aptamers, saccharides, and small molecules can be employed [98,99,151]. Despite their unique advantages, there are some concerns associated with each that should be considered first. For example, although antibodies have the most specific ligand binding, there is a risk of activating immune responses that can be mitigated, for example, by the aforementioned PEG polymers [152]. In contrast, the aptamers are relatively less specific but with much lower immunogenicity than antibodies [153,154,155]. Ultimately, a successful drug delivery must be followed by a controlled release pattern over the location and timing. Otherwise, the cargo might be diffused out before reaching the target site.

### 3.4. Drug Release

**Figure 4:**
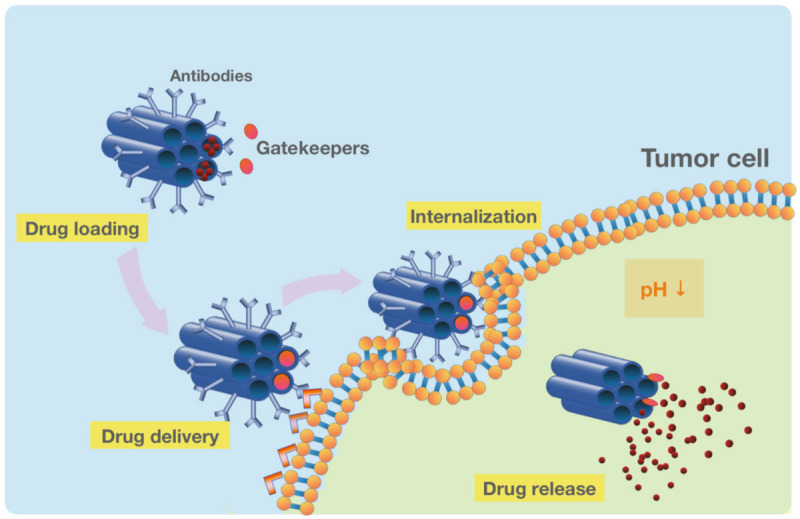
**The functionalized MSNs for the targeted drug delivery and release in tumor cells.** The pH-sensitive drug-loaded MSNs are conjugated with an antibody to target cancer cells specifically. After the delivery and internalization of MSNs, the pH-sensitive gatekeepers release the drug in response to the acidic tumor microenvironment.

The unique structure of MSNs with a network of mesoporous channels maintains drugs in a non-crystal state that favors drug dissolution [156]. Pore morphology greatly influences the drug release into the dissolution medium [125,157]. For example, the drug dissolution rate from MCM-48 with interconnected mesoporous structures is higher than MCM-41 with unconnected pores due to the reduced diffusion impediment [79]. Another important factor to consider is that the pore size has a direct effect on the dissolution rate as the larger pore size facilitates the drug release from MSNs [158]. In addition, the length of the pore channel contributes to the drug diffusion hindrance [159]. A comparison of different lengths in both SBA-15 and MCM-41 carriers exhibits a slower drug release from MSNs with longer pore channels [69,160]. Relatively few studies have demonstrated the application of MSNs for improving the dissolution of poorly water-soluble drugs owing to their tunable surface chemistry [103]. MSNs improve the hydrophobic drug solubility by reducing the lattice energy by converting their crystalline structure into an amorphous state [161,162]. However, more research focused on the development of modified MSNs for the sustained drug release [69,70]. For example, by the conjugation of functional groups to MSNs, the interaction between the cargo and the carrier is increased, leading to slower drug diffusion and sustained release [71,101,102]. To control the drug release, gatekeeper molecules such as gold NPs and bulky proteins are commonly used to block the pore access, thereby avoiding the premature delivery of the cargo. Depending on the type of gatekeepers, different internal (e.g., pH, redox state, endogenous enzyme) [163,164,165,166] and external (e.g., heat, light, sound, magnetic field) [18,167,168,169] stimuli are used to induce the opening of the pore outlets of MSNs and the drug release [123]. Upon exposure to the stimulus, these pore keepers are either degraded or bound to silica surfaces via scissile bonds without disintegrating MSNs [25]. One of the widely used stimuli for controlling the drug release in cancer cells is the pH, as these cells are more acidic compared with healthy cells [19]. In this strategy, pH-sensitive gatekeepers are bound to the surface of the MSNs by non-covalent linkage and block the drug diffusion under neutral conditions [13,14,165,170]. The acidic environment triggers the capped pores resulting in the release of the drug [171,172]. Another internal stimuli, the redox state, is also used for inducing the drug release for the cancer treatment [163]. This stimulation relies on the significant increase in the intracellular glutathione (GSH) level relative to the extracellular medium observed in most cancers. In this approach, the disulfide bonds between the redox-responsive gatekeeper and MSNs are cleaved by GSH, leading to the cap opening and subsequent drug release [163,173,174,175]. Recently, an interesting study utilized MSNs with gatekeepers responsive to both temperature and pH for a more regulated delivery of chemotherapeutics into lung cancer cells [176]. Two strategies are commonly used for the preparation of MSNs for targeted and controlled release. The first strategy involves designing a multifunctional molecule, which functions both as a gatekeeper and a targeting agent [98]. For example, in hyaluronic acid (HA)-conjugated MSNs, the HA can be utilized to work as an enzyme responsive gatekeeper as well as a targeting ligand. In this approach, HA ligand specifically binds to CD44 receptors overexpressed in cancer cells [96]. In addition, as a pore keeper, HA is degraded upon the exposure to hyaluronidase, which is abundant in the tumor microenvironment. In the second strategy, the gatekeeper is further functionalized in order to bind with a specific receptor on target cells and achieve targeted and controlled release [99]. For example, a redox responsive gatekeeper is attached to the surface of MSNs and further decorated with Arg-Gly-Asp (RGD) peptide, a tumor targeting ligand. The encapsulated drug within MSNs is released into the tumor cells after being triggered by the GSH as mentioned above [26] (Figure 4).

## 4. Update on MSN Applications in Nanomedicines

### 4.1. The Application of MSNs in Tissue Engineering

The potential of applying MSNs in the field of regenerative medicine has been extensively explored. Since the 1980s, the multidisciplinary field of regenerative medicine has been evolving with the aim of developing biological substitutes to restore, replace, or regenerate defective tissues [177]. The key components of engineered tissues include cells, growth-stimulating signals, and the scaffolds, which are polymeric biomaterials that provide structural support for cell attachment. To restore damaged tissues and organs effectively, the choice of scaffold material and its surface properties are hence crucial for efficient cellular interactions for tissue formation. However, despite the rapid advancements in various scaffolds, the commonly used scaffolds are limited in their physical and mechanical stability and may not be suitable for bearing high loads [177]. In this regard, MSNs could be applied as a simultaneous reservoir of bioactive factors and scaffolds to mimic the natural extracellular matrix (ECM) and thus improve the effectiveness of bone and vascular tissue regeneration and wound healing.

#### 4.1.1. MSN Application in Bone Tissue Engineering

With a progressively aging population, the prevalence of bone diseases, such as bone cancer, bone infection, and osteoporosis, has led to increasing demand for bone tissue regeneration. For this reason, most recent research works on the application of MSNs in tissue engineering have substantially focused on bone tissue regeneration. However, current treatments carry several drawbacks, including poor bioavailability and the need for using higher dosages that may cause severe complications, such as the generation of drug-resistant bacteria and persistent bacterial biofilms [178,179]. One of the applications of MSNs in cell-directed drug delivery inside 3D scaffolds was the attachment of MSNs loaded with estradiol and coated with polyelectrolyte multilayers of gelatin/chitosan (E2-MSN@PEM) on titanium substrates [180]. The modified surface improved the properties of this biomaterial for prolonged intracellular gene delivery in bone tissue engineering and hence the bio-behavior of osteoclasts to maintain bone homeostasis.

Other research groups have also developed multifunctional hybrid nanofibrous scaffolds that could load and deliver drugs at high levels more efficiently and over long periods. For example, the hybrid scaffolds of polycaprolactone shelled with mesoporous silica (PCL@MS) were developed to provide a 3D microenvironment that allows the slow and sustained diffusion-controlled release of the PCL nanofiber. These scaffolds can thus effectively control and stimulate the desired cellular responses and subsequent tissue reactions for bone regeneration [181]. In another study, a composite scaffold of PLLA incorporated dexamethasone loaded MSNs coated with chitosan (MSNs-DEX@CS/PLLA) were prepared to enhance the osteogenic potential of pure poly-l-lactic acid (PLLA) scaffolds [182]. These MSN-based nanocarriers have a conductive surface that can improve the scaffold’s osteogenic potential, resulting in better control in the pH-sensitive delivery of dexamethasone at the site of implantation. Another example of a hybrid nanofibrous scaffold delivered with MSNs is the peptide-laden MSNs possessing ordered pores loaded with bone-forming peptides to enhance the controlled release of osteogenic factors [131]. These peptide-modified MSNs with increased surface area and pore volume accelerated the kinetics of scaffold deposition, making them a potential material for bone repairing applications [131].

In addition to the improvement of the delivery scaffolds for suitable growth factors, MSNs have also been applied to stimulate osteogenic [183,184,185,186,187], myogenic [188,189], and angiogenic differentiation of bone mesenchymal stem cells (BMSCs). For example, Shi et al. used dimethyloxalylglycine-loaded MSNs (D-MSNs) with a uniform sphere size of ~90 nm and mesopores of ~2.7 nm [190]. These D-MSNs are able to release silicon ions and hypoxia-inducing drugs that can promote human bone marrow mesenchymal stem cells (hBMSCs) to undergo osteogenic and angiogenic differentiations. For treating large bone defects, the demineralized bone matrix (DBM)-MSN/152RM scaffolds also have a strong capacity in inhibiting PTP1B Y152 phosphorylation, promoting MSCs differentiation in bone formation [191]. In a separate study, Zhou et al. synthesized BMP-2 peptide-functionalized and dexamethasone-loaded MSNs (DEX@MSNs-pep) as a delivery system in stimulating in vitro osteogenic differentiation [133]. Likewise, Luo et al. synthesized MSNs incorporated with bone-forming peptides that contained the cell differentiation-promoting alginate hydrogel (RA) (pep@MSNs-RA). They demonstrated that this pep@MSNs-RA could promote osteogenic differentiation and the survival of human MSCs. While these molecules are highly effective in regulating the viability and growth of BMSCs and accelerating osteogenesis, one of the major limitations is their burst release. Hence, incorporating a scaffolding system that absorbed these osteogenesis molecules, such as N-acetylcysteine (NAC), into surface-modified MSNs (NAC@MSNs), has been shown to act as a controlled drug delivery vehicle [192]. This vesicle enables the sustained and controlled release of NAC and promotes the osteogenic differentiation of rat bone marrow mesenchymal stem cells (rBMSCs). In another study, MSNs that were functionalized with calcium, phosphate, and dexamethasone (MSNCaPDex) were combined with organic gelatin methacrylate (GelMA) [193]. The resulting products were further used as a bioink for the 3D-bioprinting of osteogenic constructs. These constructs exhibited osteogenic differentiation biomarkers without further requirement of neither biochemical nor mechanical stimuli, showing their efficient abilities in inducing hBMSC differentiation for bone tissue repair.

MSNs were also applied to deliver siRNA molecules for treating both bone cancer and osteoporosis. Kim et al. first developed a siRNA delivery system by introducing amine-functionalized mesoporous bioactive glass nanospheres (MBG) to deliver siRNA that can knock down the RANK gene and further suppress osteoclast genesis [194]. Besides, PEI-coated MSNs (MSNs@PEI) have also been used in a similar way to load osteostatin (osteogenic peptides) and siRNA [195]. The co-delivery of both therapeutic reagents resulted in a synergistic effect on SOST gene knockdown and osteogenesis in ovariectomized mice. These data suggested the MSN-mediated co-delivery of osteostatin and siRNA as an alternative approach for osteoporosis therapy. Furthermore, Shi et al. synthesized Cu-containing MSNs (Cu-MSNs) as delivery vehicles with reported sustained cargo release and controlled degradability. The uptake of Cu-MSNs by immune cells led to the modulation of the immune microenvironment via inducing proinflammatory cytokines, osteogenic/angiogenic agents, and suppressing osteoclastogenic factors. These lead to the osteogenic differentiation of BMSCs through activating the Oncostatin M (OSM) signaling pathway [196].

Another notable cause of concern in osteoporosis therapy is the chronic inflammatory conditions due to estrogen deficiency among postmenopausal women. Under such conditions, T cells are activated to elevate the proinflammatory cytokines, which impairs the osteogenic differentiation capabilities of BMSCs. To eliminate excessive activated T cells, T cell-depleting MSNs (TDNs) consisting of monocyte chemotactic protein-1 (MCP-1) and Fas-ligand (FasL) were used to re-establish immune homeostasis in this disorder. Additionally, MSNs have also shown the potential to repair large bone defects. Jia et al. synthesized mesoporous silica-coated magnetic (Fe_3_O_4_) nanoparticles (M-MSNs) and demonstrated their potential for accelerating osteogenic differentiation in the model of distraction osteogenesis (DO) [197].

Osteoarthritis (OA) is one of the most explored diseases in regenerative medicine as it can be tackled by two approaches, either by engineering replacements for damaged tissues, injecting stem cells, or blood products into the body to repair itself. However, current treatment options are limited and hindered by inflammatory irritation and cartilage degradation. To improve the treatment efficacy, MSNs have thus been used to load and directly deliver anti-inflammatory drugs into the joint capsule to sustain their effects. Zhao et al. generated visible light-responsive dual-functional biodegradable MSNs that enhanced the local release of the anti-inflammatory drug diclofenac sodium, thereby improving joint lubrication in the treatment of OA [198].

Nerve fibers regeneration is another important aspect of bone formation. Since inadequate innervation in the bone defect area can significantly hinder nutrient metabolism in the bone defect area, the regeneration process is delayed, and bone quality is decreased. For this reason, Lei et al. developed an injectable thermosensitive MSN-embedded core-shell structured poly(ethylene glycol)-*b*-poly(lactic-*co*-glycolic acid)-*b*-poly(*N*-isopropylacrylamide (PEG-PLGA-PNIPAM) hydrogel for long-term co-delivery of aspirin (ASP) and microRNA-222 (miR222/MSN/ASP hydrogel). Aspirin and miR222, release from the miR222/MSN/ASP hydrogel, can stimulate bone formation and the differentiation of human BMSCs into neural-like cells. In a rat bone defect model, injection of miR222/MSN/ASP hydrogel led to both neurogenesis and enhanced bone formation, indicating miR222/MSN/ASP hydrogel as a promising material for innervated bone tissue engineering [199]. In another study, Mehrasa et al. fabricated aligned poly lactic-co-glycolic acid (PLGA) and PLGA/gelatin nanofibrous scaffolds embedded with MSNs and demonstrated their utility to improve the tensile performance of the scaffolds, which eventually contributed to the improvement of neuronal cell attachment and proliferation [200]. Together, these findings highlighted the potential of these MSN-based aligned nanofibrous scaffolds in nerve regeneration and outgrowth.

#### 4.1.2. MSN Application in Vascular Tissue Engineering

Unlike the burgeoning development in bone tissue engineering, the applications of MSNs for vascular tissue engineering have been explored much less. Besides the aforementioned applications of MSNs in angiogenic differentiation of stem cells, MSNs were also utilized as a carrier to load cargoes for stimulating vascularization in tissues with minimizing risks of thrombosis and occlusion. For example, Wu et al. mobilized heparin-loaded MSNs onto silicon substrates and assessed the anticoagulant effect of the modified silicone films [201]. The results demonstrated that the sustained release of heparin by MSNs effectively prevents the adhesion of platelets and blood cells, implying the excellent biocompatibility of these functionalized MSNs. Shokry et al. also developed bioactive scaffolds that support vascularization by promoting tissue growth and functions [202]. The hybrid PLA–PANI–MSN scaffold is composed of electro-spun polylactic acid (PLA) polyaniline (PANI) fibers with controllable fiber orientation and scaffold thickness carrying physisorped MSNs within the fiber matrix. This scaffold has proven to vascularize in vivo upon transplantation in chicken embryos and hence support locally retained cell-targeted tissue formation without adverse effects on the homeostasis of adjacent tissues.

An example of drug-loaded MSNs incorporated into the scaffolds for vascular implants is the salvianolic acid B (SAB)-loaded MSNs (SAB/MSNs) [203]. SAB has the potential to promote the proliferation and migration of endothelial cells and can be released by the MSNs in a controlled and sustained manner. These SAB-MSNs exhibit strong anticoagulation and good blood compatibility for enhancing human umbilical vein endothelial cell (HUVEC) growth. It sheds light on a promising way to promote rapid re-endothelialization of artificial vascular grafts without developing intima hyperplasia and acute thrombosis. In another study, Guo et al. incorporated Salvianic acid (SA)-loaded MSNs into gelatin/polyurethane bilayered small-diameter tubular scaffold [204]. The poly(ester-urethane) urea (C-PEEUU) nanofibers were subsequently electrospun outside the SAL@MSNs/Gelatin vascular scaffold, strengthening its spongy matrix with sustained release of drug and good mechanical properties. These bi-layered vascular scaffolds (SAL@MSNs/Gelatin/C-PEEUU) have promising prospects for vascular tissue engineering applications.

To stimulate vascularization and osteogenesis simultaneously, MSNs can also act as an effective dual delivery system. For example, Wang et al. synthesized alendronate@MSNs (ALN@MSNs) to co-deliver alendronate (ALN) and silicate for the synergistic effect on bone remodeling [205]. ALN suppressed the bone resorption while silicate improved vascularization and bone calcification, contributing to the bone-forming process. In another study, Ma et al. constructed 3D-printed silver nanoparticles (P-AG-MSNs) loaded with platelet-derived growth factor BB (PDGF-BB) that could activate the alkaline phosphatase (ALP) activity of bone-relate genes [206]. As a result of the increased gene expression, these P-AG-MSNs could stimulate the osteogenesis and angiogenesis of BMSCs. In another study, Kim et al. (2016) reported that incorporating vascular endothelial growth factor (VEGF)-loaded MSNs into type I collagen sponge could produce the collagen/MSN/VEGF (CMV) scaffold [207]. These CMV scaffolds were able to significantly increase the number of blood vessel complexes through their VEGF-releasing capacity for effective vascularization.

For myocardial infarction, the current therapeutic strategies are blurred, and a strategy to combat excessive inflammation while enhancing angiogenesis is necessary. To achieve a synergistic anti-inflammatory and proangiogenic effects, Li et al. developed a controllable MSN-based drug delivery system in which anti-inflammatory agent microRNA-21-5p loaded MSNs were encapsulated into an injectable hydrogel matrix (Gel@MSN/miR-21-5p). The released MSN complexes notably inhibited inflammatory responses in a porcine model of myocardial infarction [208,209].

In addition, the delivery of miR-21-5p to vascular endothelial cells further induced local neovascularization and rescued at-risk cardiomyocytes. The delivery of injectable hydrogel with MSNs/miR-21-5p has shown the promising therapeutic potential that can restore myocardial functions in myocardial infarction.

#### 4.1.3. MSN Application in Wound Healing and Antibacterial Effects

Besides tissue regeneration, wound healing also plays an equally important role in restoring the tissue integrity of diseased and wounded skins. However, the engineered materials for the synergistic promotion of tissue engineering and wound healing remain elusive. Wound healing requires the scaffold’s mechanical support and simultaneously needs the improved angiogenesis property to accelerate the healing process [210,211,212,213]. Most applications of MSNs that were designed for this purpose followed the study conducted by Ren et al. in 2018. They prepared the aligned porous poly (l-lactic acid) (PlLA) electrospun fibrous membranes containing dimethyloxalylglycine (DMOG)-loaded MSNs (D-MSN) for promoting diabetic wound healing [214]. Notably, the D-MSN particles were distributed on the aligned nanofibrous surface membrane. The DMOG and Si ions could be controllably and simultaneously released from the nanopores on the fibers. These released components could synergistically promote the adhesion, proliferation, migration, and angiogenetic differentiation of HUVECs at the wound sites, thereby accelerating wound healing. Following the same concepts, various types of MSNs [214,215], such as MSN@CS-HCA developed by Chen et al. (2019) [216], tannic acid (TA)-loaded MSNs by Wang et al. (2018) [217], and GQDs@HMSN(EM) by Wang et al. (2020) [218], have been synthesized for a similar purpose of accelerating wound healing.

**Figure 5:**
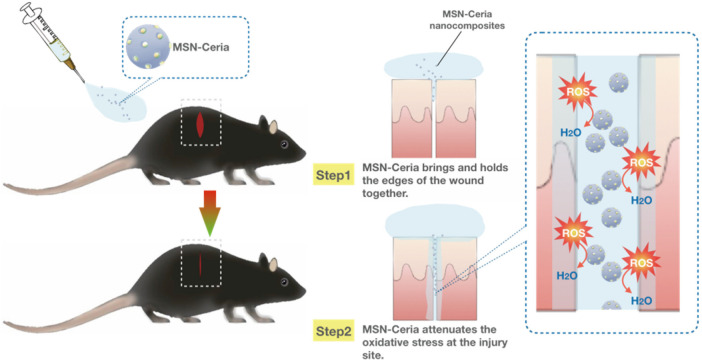
**MSN-Ceria as a ROS-scavenging tissue adhesive nanocomposite in wound healing.** MSN-Ceria carries both adhesion properties and ROS-scavenging potential. MSN-Ceria holds the edges of the wound together, followed by the attenuation of ROS generation at the injury site, leading to enhanced skin wound healing.

One of the limitations of wound healing is the overproduction of reactive oxygen species (ROS) at wound sites that may inhibit the repair process by triggering harmful reactions such as cellular senescence, fibrotic scarring, and inflammation [219,220]. Therefore, ROS-modulating agents with the ability to reduce oxidative damage at injured sites become a promising alternative for wound repair and regeneration process. Wu et al. used ceria nanocrystal-decorated MSNs (MSN-Ceria) as a ROS-scavenging tissue adhesive nanocomposite in wound healing, owing to the combination of MSN’s unique adhesion property and the ROS-reducing capacity of Ceria nanocrystals. After applying onto the wound, the MSN-Ceria initially holds the edges of the wound together and then attenuates the generation of oxidative stress at the injury site (Figure 5). Collectively, MSN-Ceria carries both strong adhesion strength and remarkable ROS-scavenging potential, leading to accelerated skin wound healing [221].

Despite the harmful effects of ROS, several reports indicated the beneficial role of ROS in arresting bacterial growth. Bacterial infection is a major concern during the wound healing process. To combat bacterial infections, MSNs have been utilized in generating ROS to kill bacteria and improve wound healing. For example, cAg/AgBr-loaded MSNs (Ag/AgBr/MSNs) have been shown to enhance their photocatalytic effect by the production of ROS, resulting in bacterial growth arrest and bacterial membrane damage [222,223]. The gradual and sustained release of Ag/AgBr/MSNs exhibit remarkable photocatalytic ability with long-term effect through generating electron-hole pairs after light absorption. Ag/AgBr/MSNs demonstrated a bacterial killing efficiency of 95.62% and 99.99% against *Staphylococcus aureus* and *Escherichia coli,* respectively, under simulated solar light irradiation. The gradual release of Ag+ stimulated the immune response to produce neutrophils and leukocytes, leading to the improvement of the wound healing process [222,223]. These findings highlighted the potential of Ag/AgBr/MSNs in preventing bacterial infection during wound healing. Besides, modified CuS MSNs have also exhibited high antibacterial efficacy of 99.80% and 99.94% against *Staphylococcus aureus* and *Escherichia coli*, respectively. The remarkable anti-bacterial effect of CuS MSNs was attributed to the combined effects of hyperthermia, radial oxygen species, and the release of copper ions under NIR irradiation. The released copper ions further stimulated fibroblast proliferation. Collectively, the combined effects of CuS MSNs can promote both antibacterial effects and skin tissue regeneration under defined conditions [224].

The hemorrhage control and effective anti-bacterial treatment via covalent conjugation and electrostatic adsorption have also been widely explored with fabricated biomaterials. For example, Lian et al. synthesized a novel bi-layered membrane, comprising of a loose layer (LL) of conjugated poly (lactic-co-glycolic acid) (PLGA)/gelatin nanofibers incorporated with the dexamethasone drugs-loaded MSNs (DEX@MSNs), as well as another dense layer (DL) of PLGA nanofibers loaded with the antibiotic doxycycline hyclate (DCH) [225]. While the LL membrane exhibited enhanced osteogenic capacity and calcium deposition of rat BMSCs due to the increased cell infiltration, the DL membrane acted as a barrier for antibacterial effects. This demonstrated the combined osteogenic and antibacterial properties of the sustained release in this bi-layered composite membrane for guided bone regeneration applications. In another study, Chen et al. constructed MSNs with double folic acid (FA) and calcium phosphate (CaP) via electrostatic interaction and biomineralization to effectively inhibit drug-resistant bacteria without producing drug resistance [226]. Amp-MSN@FA@CaP@FA could reduce the mortality caused by the drug-resistant *E. coli* via the sustained drug release and promotion of wound healing. Consequently, these resulted in increased intake and retention time of antibiotics, accelerating the healing process. In another study, Encinas et al. designed similar MSN-based systems focusing on their host body recognition for adequate actuation and biocompatibility [227]. These silane-modified MSNs showed improved surface properties and low-fouling functionalization in comparison to PEGylated MSNs. The zwitterionic MSNs (zMSNs) reduced up to 70–90% of protein adsorption and 60% of cellular uptake, signifying a promising future for developing these novel antimicrobial mixed charge zMSNs.

The range of MSN applications in tissue engineering has expanded rapidly with advancements in the biomaterials. By optimizing their mechanical properties and biocompatibilities, MSNs offer a highly efficient platform for targeted and sustained delivery of drugs in the 3D scaffolds. The potential for MSN applications in tissue engineering is endless, from enhancing bone, nerve, and vascular tissues regeneration to facilitating antibacterial wound healing. With continued developments, the incorporation of MSNs in 3D-printed structures would offer abundant opportunities for the formation of an even wider variety of specific functional tissues, such as customizable scaffolds.

### 4.2. MSN Applications in Bioimaging

Besides effective treatment, early and precise diagnosis is another goal of continued efforts in biomedical research [228]. There are various existing theragnostic imaging techniques, including optical imaging, magnetic resonance imaging (MRI), positron emission tomography (PET), computed tomography (CT), ultrasound imaging, and multimodal imaging. To date, a broad range of nanomaterials has been developed for these imaging techniques, many of which offer notable advantages over conventional molecular methods. These nanomaterials include superparamagnetic iron oxide nanoparticles (SPIONs) approved by FDA for MRI contrast acquisition [229], gold nanoparticles used for CT [230,231,232], and inorganic semiconducting NPs, such as quantum dots (QDs) or upconverting nanoparticles (UCNPs) used for narrow and tunable emission spectra in optical imaging [233,234,235,236]. However, some drawbacks of these imaging agents, such as difficulties in surface modification and physiological instability upon in vivo administration, need to be overcome. Although surface functionalization of many NPs faces extremely challenging barriers, such as the expulsion by the immune system or high osmotic pressure of cells, NPs have been widely exploited as vehicles for delivering imaging agents [237,238,239,240]. In addition, nano-sensors also tend to be inactivated due to the invasion and adsorption by serum proteins in a process known as biofouling [241,242]. Considering the unique advantages of MSNs, as described in Section 2, the integration of MSNs and nanomaterial-based imaging agents has proven to be highly promising. MSNs have been shown to be suitable for long-term quantitative imaging at low doses and safely cleared from the body following detection [243]. Besides, MSN-supported imaging agents have been developed with preserved essential imaging properties [244]. MSNs have demonstrated their potential to remain chemically stable in a colloidal suspension on various physiological conditions (i.e., ionic solvent, pH, and temperature) while providing a good imaging contrast (i.e., high signal-to-noise ratio) for various imaging modalities [28,245,246].

#### 4.2.1. MSN Application in Optical Imaging

Optical imaging techniques are among the major imaging methods used for clinical applications, offering an easy and simultaneous visualization of subcellular structures. Fluorescence and bioluminescence are the basis of most optical imaging approaches. Through labeling and direct data interpretation, they can analyze multiple subjects with a high spatiotemporal resolution that breaks the diffraction limit. Acquiring high-quality optical images by the probing system greatly relies on adequate source of photons [247,248]. One way to achieve this is to secure the fluorescence probes within MSNs owing to their tunable pore size and high pore volume to the surface area that provides high loading capacity. For example, Lee et al. encapsulated the indocyanine green (ICG) into MSNs [249] and successfully fabricated probes using the trimethylammonium groups-modified MSNs (MSN-TA-ICG) that enhanced the fluorescence photon count of ICG. Similarly, Sreejith et al. synthesized GO-MSNs for optical imaging probes. They loaded squaraine (zwitterionic IR dyes) onto the MSN’s pores and wrapped the pores with graphene oxide to prevent unwanted nucleophilic substitution and leakages of the dye, increasing the efficiency of the imaging probe [250]. In another study, HMSNs were encapsulated within the polymer *N*,*N*-diphenyl-4-(4-(1,2,2-triphenylvinyl)styryl) aniline (PTPA) that exhibited putative aggregation-induced emission (AIE) properties. In this design, the surface of HMSNs were functionalized with an anti-EpCAM aptamer. The aptamer-functionalized HMSNs delivered the fluorescent polymers to the hepatocarcinoma Huh-7 cells with excellent biocompatibility and specificity. MSNs could effectively internalize by target cells, demonstrating aptamer-functionalized HMSNs as promising candidates for targeted cellular imaging applications [251].

Additionally, the fluorescent properties of quantum dots (QDs) have been extensively explored for bioimaging [235,252]. The fluorescence wavelength shifts from blue to red as the particles’ size increasing, enabling QDs of the same material at different sizes to cover almost the complete visible color spectrum and to precisely control fluorescence properties. However, many CdSe/ZnS and ZnCdSe/ZnS (core/shell type) QDs are hydrophobic and MSNs have been used to improve their solubility in water. For example, QDs, ibuprofen drug, and magnetite nanocrystals simultaneously embedded into mesoporous silica spheres (M/GQD-MSS) exhibited superparamagnetic characteristics and water dispensability that are both desirable for their applications in drugs and genes delivery [253]. QDs and magnetic iron oxide nanocrystals loaded into MSNs have also been used to fabricate a dual-function probe that could be assigned for the magnetic capture, enrichment of biological targets, and simultaneously for optical encoding [254]. Furthermore, Hao et al. developed a novel microfluidic synthesis strategy with well-controlled physicochemical properties and used it for developing hollow spherical silica (HSS). The HSS was then assembled with different functional small-sized NPs, including QDs, silver nanoparticles, and magnetic nanoparticles, to simultaneously acquire specific magnetic, fluorescent, or catalytic properties [255]. This technology provides the intracellular and cell-specific delivery of imaging agents which are expected to contribute to clinical applications in the future.

Compared with visible fluorescence imaging, using the near-infrared (NIR) spectrum in fluorescence imaging can greatly improve imaging resolution and tissue penetration depths by reducing photon scattering and background noises [256,257,258]. Therefore, in vivo NIR fluorescence imaging has been a rapidly advancing imaging modality, including the NIR fluorescence imaging with the introduction of MSNs. For example, Lin et al. synthesized mSiO2@Zn0.6Ca0.4Ga2O4:Cr^3+^,Yb^3+^ (mSiO2@ZCGO) nanoparticles that carry a regular morphology with a relatively uniform size of about 69 nm. Remarkably, these nanocrystals exhibited persistent luminescent properties in multiple NIR windows, i.e., the first infrared widow at ~696 nm for Cr^3+^ emission and the second infrared widow at ~1000 nm for Yb^3+^ emission [259]. These MSNs also showed increased penetration depth and improved long-term imaging, suggesting a great promise of mSiO2@ZCGO for deep-tissue NIR fluorescence bioimaging applications. Besides, the in vivo fate of nanomaterials may differ depending on their administration routes. For example, most nanomaterials aggregate into the reticuloendothelial system after intravenous injection. In the oral route, nanomaterials can be destroyed by gastric acid after oral administration. These limitations drove researchers to develop novel nanomaterials that can retain their efficacy after passing various administration routes. Wang et al. constructed bacteria bioinspired NPs by coating lactobacillus reuteri biofilm (LRM) on the surface of trackable zinc gallogermanate (ZGGO) mesoporous silica (ZGGO@SiO2@LRM). These NPs enabled efficient local digestion of gastric acid and targeted release of the chemotherapy drug 5-FU to colorectum, which lasted for more than 24 h after intragastric administration [260]. In vivo experiments also demonstrated that the number of tumors per mouse decreased by about half, shedding light on the application potential of NIR-persistent emission of ZGGO@SiO2@LRM in targeted delivery of oral drugs. Additionally, the controlled synthesis of mesoporous silica-coated UCNPs using NIR (808 nm)-responsive diarylethene (DAE) photochromic switches has shown enhanced and more controlled release of singlet oxygen both in vivo and in vitro [261]. These UCNPs are capable of upconverting NIR light into tunable shorter-wavelength UV and visible light on demand, emerging as a promising platform to enable light delivery into deep tissues for various bioimaging applications.

In addition to MSNs applications in fluorescence imaging, they have also been used to deliver molecules with phosphorescent properties. Ultralong organic phosphorescence (UOP) has been widely explored as a superior alternative to fluorescence since it allows for longer luminescence after removing external excitation light due to the higher excited energy levels of electrons. However, like the fluorescence molecules, these phosphorescence molecules are hydrophobic, which limits their biological applications. You et al. used a facile and versatile approach to develop hydrophilic phosphorescent phosphors (HPPs) by loading ultralong organic phosphors into HMSNs. The resulting HPPs showed low cytotoxicity after their internalization by Hela cells [262]. The afterglow bioimaging of HPPs in the subcutaneous region of nude mice exhibited sharp intensity peaks and eliminated the autofluorescence interference with a high signal-to-noise ratio. Overall, this HMSN-based approach offers considerable potential for in vivo phosphorescence imaging.

#### 4.2.2. MSN Applications in Magnetic Resonance Imaging and Positron Emission Tomography

As an effective and widely used biomedical tool, magnetic resonance imaging (MRI) efficiently and non-invasively provides three-dimensional anatomic and functional data with high resolution. MSN-based contrast agents for MRI have been shown to exhibit increased sensitivity. This is due to the MSNs’ large specific surfaces that carry more payloads of the active magnetic centers [263,264]. Moreover, the silica mesoporous structure provides easy access to the magnetic centers, allowing MSNs to be functionalized with targeted ligands and efficiently conducted to damaged tissue [265,266,267,268,269]. For example, multifunctional MSNs with a magnetic core were developed by Chen et al. for magnetic-enhanced tumor-targeted MRI and therapy. First, the gatekeeper β-cyclodextrin (β-CD) was immobilized on the surface of MSNs via the linker of platinum prodrug (IV). The introduction of Arg-Gly-Asp (RGD) peptide ligand onto the gatekeeper β-CD allowed these MSNs to meet their cancer-targeting purpose. After endocytosis by the cancer cells, platinum (IV) prodrug was converted to the active platinum(II) drug in the cancer cell microenvironment. Subsequently, the detachment of gatekeeper β-CD triggered the in situ release of doxorubicin to achieve the tumor-suppressing efficacy. With the aid of external magnetic fields, doxorubicin-loaded MSNs exhibited a magnetically enhanced accumulation at the cancer site and accurately inhibited the cancer growth with minimal side effects [270]. Notably, in addition to the aforementioned factors such as nanoparticle shape, pore size, and surface area, a few studies have also shown that MSNs’ performance for MRI can be enhanced by forming the MSN-metal complexes with different metal ions, such as Al-MCM-41, Mn-MCM-41, and Fe-MCM-41 [153,154,271,272,273,274]. MSNs have also been employed in positron emission tomography (PET). Jeong et al. designed a PET imaging protocol for in vivo macrophage cell tracking using aza-dibenzocyclooctyne-tethered PEGylated MSNs (DBCO-MSNs) with F-18-labeled azide-radiotracer. This approach allowed the successful visualization of macrophage migration into the tumor sites and atherosclerotic plaques in tumor-bearing and ApoE^-^/_−_ mouse models, respectively. Notably, the results of tissue radioactivity distribution were consistent with PET findings. This in vivo cell tracking approach exhibited ideal performance in short-term and relatively long-term monitoring of cell viability [275]. In another study, Jung et al. showed that single human breast cancer cells loaded with MSNs concentrating the ^68^Ga radioisotope can be tracked in real-time after the injection into immunodeficient mice. Therefore, this MSN-based single-cell tracking method could be used to determine the kinetics of tumor cell trafficking and arrest in the early phase of the metastatic cascade through the body [145].

#### 4.2.3. MSN Application in Multi-Modal Imaging

**Figure 6:**
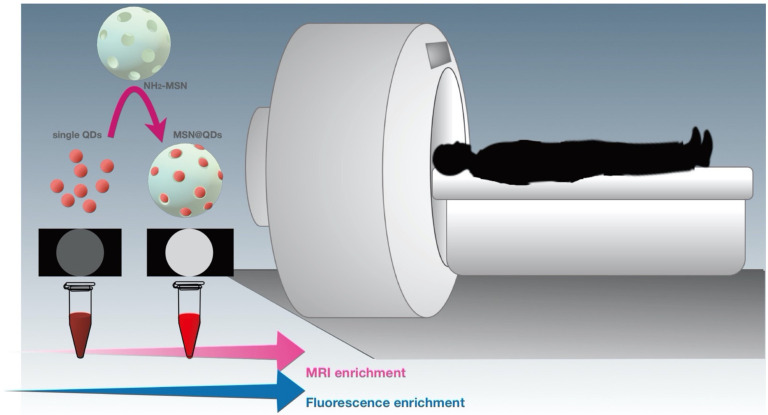
**MSNs as a dual imaging probe in bioimaging.** Schematic illustration of MSNs as a dual-imaging probe to synergistically enhance MRI and fluorescence in bioimaging applications.

While imaging techniques discussed in the preceding sections have their unique advantages, they also come with innate limitations, including low resolution, low penetration depth, and poor sensitivity. To maximize their accuracy and applicability in specific cellular sites, combining two imaging techniques, such as the combination of ultrasound imaging and MRI or the combination of optical imaging with either MRI, PET, or CT, have been shown as an alternative and powerful strategy [276,277,278]. For example, dual-imaging probes combining NIR-enhanced fluorescence imaging and MRI have emerged as a popular choice for adjuvant therapy and diagnosis over the past decade. Importantly, MSNs have also been utilized in the preparation of such probes [267,279]. In particular, He et al. doped MSNs with an aggregation-induced emission (AIE) dye and Gd^3+^ through a direct sol-gel method. The resulting MSNs emitted strong red fluorescence with a maximum emission wavelength of 669 nm and could be utilized as effective fluorescence probes for fluorescence microscopy imaging. Notably, the introduction of Gd^3+^ could improve the contrast of MRI, making these MSNs ideal contrast agents in contrast-enhanced MRI [280]. Another example for multimodal imaging is the Mn-doped quantum dots (QDs), such as the Mn-doped ZnS (ZnSe) QDs, which possess unique fluorescent and magnetic properties. However, the optimal Mn^2+^ doping concentration for maximizing the fluorescence of QDs is relatively low for MRI imaging. Given that Mn-doped ZnSe QDs carry large stokes shift fluorescence and magnetic resonance, Zhou et al. designed an enrichment strategy with MSN loading to construct a highly luminescent/paramagnetism Mn-doped ZnS (ZnSe) QDs assembly (MSN@QDs) that allow the improvement of MRI/optical dual-model imaging. Carboxyl-functionalized Mn-doped QDs were loaded into the large pores within amine-functionalized MSNs (NH_2_) via the 1-(3-dimethylamino-propyl)-3-ethylcarbodiimide hydrochloride/N-hydroxysuccinimide coupling, resulting in the MSN@QDs. Through the assistance of large pore MSNs, local Mn^2+^ increased largely and therefore improved the MRI sensitivity [266] (Figure 6). In a separate study, Wu et al. loaded NIR fluorescence dye IR820 into MSNs conjugated with PP_1_ peptide to specifically target and quantify macrophage enrichment in atherosclerotic plaques in ApoE^-^/_−_ mice. Taking advantage of the distinctive properties of MSNs allows precise imaging of high-risk plaque, promoting the treatment and the prevention of vascular pathema [279].

While MRI is an essential imaging modality for clinical diagnosis, MRI-guided high-intensity focused ultrasound (MRgHIFU) has recently emerged as a powerful technology for targeted therapy in cancerous tissues. The thermal damage during drug delivery applications of MRgHIFU is an important matter of concern. Therefore, establishing a cellular environment with a physiological safe temperature range can help reduce the possibility of such damage to surrounding tissues. Cheng et al. developed an MRI-guided high-intensity focused ultrasound (MRgHIFU)-responsive MSN platform that could release the imaging agent cargo molecules gadopentetate dimeglumine (Gd(DTPA)2-) upon the stimulation by MRgHIFU. These MSNs allowed the biodistribution of nanocarriers of MRgHIFU-stimulated cargo to be visualized, demonstrating its potential in MRI image-guided theragnostic applications [265].

Besides the application of MSNs in fluorescence imaging, MSNs, such as Au nanoclusters (AuNCs)-loaded MSNs, have also been utilized in computed tomography (CT) imaging. For example, an enhanced NIR-enhanced fluorescence/CT dual-modal imaging probe was constructed, enabling the AuNCs-loaded MSNs (MSN@AuNCs) to detect CAL-27, ACC-2, and SCC-25 oral carcinoma cell lines [233]. Compared with the simple AuNCs, MSN@AuNCs exhibited improved NIR fluorescence/CT performance and revealed a remarkable potential to create sensitive dual-modal imaging probes for the diagnosis and treatment of diseases.

Trimodal synergistic imaging, including a combination of optical imaging, MRI and CT, or optical imaging, MRI and PET, has attracted increasing attention in various cancer therapies. To improve the efficiency of such synergistic therapies and multifunctional imaging, developing a smart tumor microenvironment (TME) for guided delivery is indispensable. In this regard, Fe–Mn layered double hydroxides (FeMn-LDH) was used as an effective photothermal nanocarrier for chlorin e6 (Ce6)-coated UCNPs [281]. As Ce6 imaging agents were delivered and released into HeLa cells, these nanocarriers could induce high resolution MRI guidance to enhance the efficacy of chemodynamic therapy, demonstrating their potential in cancer theragnostic applications. Similarly, doxorubicin (DOX)-loaded mesoporous silica-coated gold cube-in-cubes core/shell nanocomposites with anchored Mn-Cdots were synthesized to improve the therapeutic efficacy of DOX and render them multiplexed bioimaging agents consisting of photothermal, fluorescence, and MRI [282]. These RGD-CCmMC/DOX nanocomposites exhibited improved heat/pH-sensitive drug release to specific tumor sites with precise control. These data indicated that RGD-CCmMC/DOX nanocomposites could function as a therapeutic agent by combining chemo-phototherapy and multimodal bioimaging techniques. In another study, Sanchez et al. constructed a novel Janus nanoplatform by combining the Fe_3_O_4_NPs/mesoporous silica core@shell face with the Au nanoparticle face. Using a fibrosarcoma-bearing mouse model, this hybrid nanomaterial exhibited an excellent potential in multimodal imaging by magnetic resonance (Fe_3_O_4_ core), CT (AuNP face), and fluorescent tracking (fluorescent dye loading) [283]. These findings suggested that this hybrid nanomaterial may open new avenues for anti-cancer therapy and concomitant visualization of cancer tissues.

Overall, MSNs have been shown to be a promising delivery platform for imaging agents due to their high surface area, controllable mesopores, and facile surface functionalization. With further improvement in these noninvasive bioimaging technologies, MSN-aided systems can contribute to early and effective diagnosis in clinical applications. The further development of these noninvasive bioimaging technologies allows MSN-aided systems to contribute to early and effective diagnosis in clinical applications.

### 4.3. MSN Application in Stem Cell Research

#### 4.3.1. MSN Application in Stem Cell Maintenance and Differentiation

MSNs have been increasingly employed as an ideal candidate for stem cell therapies, both for delivering bioactive factors and by incorporation into the polymer-based scaffold to improve its mechanical properties [188,284,285]. In these studies, MSNs contribute to stem cell maintenance and differentiation by altering the properties of scaffold or providing signals required for stem cell function [286]. The functional communication and interaction between biomaterial and nanoparticles play an indispensable role in successful tissue engineering [188,287]. Therefore, examining the effect of NPs characteristics (e.g., size, morphology, modifications) on stem cell maintenance and differentiation is a key step to achieving expected results [288,289]. For example, Argentati et al. showed that star-shaped MSNs conjugated with different functional groups have different impacts on hBMSCs and adipose stem cells (hASCs) morphology and functionality [290]. In this design, MSN pores were loaded with bone-forming peptide-1 (BFP-1), an inducer of osteogenic differentiation. The resulting pep-MSNs were encapsulated into the arginine-glycine-aspartic acid (RGD)-treated alginate hydrogel (RA) to obtain pep-MSNs-RA. The binding of RGD peptides to receptors on the cell surface supports the viability and proliferation of HMSNs induced by BFP-1, before exposing them to the osteogenic differentiation. This design allows a stepwise manner in proliferation and differentiation, thereby leading to better mimicry of the developmental process. Another example for the utilization of MSNs for stem cell differentiation was reported by Hosseinpour et al. [291]. In this study, MSNs coated with polyethylenimine (MSNs-PEI) were utilized to deliver Rattus norvegicus (rno)-miRNA-26a-5p into rBMSCs and promote their differentiation to osteoblast. The efficient delivery of the miRNA was validated by significant increases in osteogenic differentiation, matrix deposition, and mineralization at week 3 of post- transfection. In addition, the lyophilized MSNs-PEI delivery system was reported to be stable even after 6 months of storage. Very few studies investigated how the interaction between nanobiomaterials and the immune system can contribute to the regenerative capacity of bone marrow stromal stem cells. To address this question, Shi et al. incorporated therapeutic ion europium (Eu) into MSNs, generating a uniform spherical-shaped morphology. Eu can functionally mimic calcium ions and be used for treating osteoporosis disorder [292]. The EU-MSNs could induce the release of pro-inflammatory signal, followed by the osteogenic differentiation of bone marrow stromal cells indicating the link between the early inflammation and osteogenesis in bone regeneration process [293].

**Figure 7:**
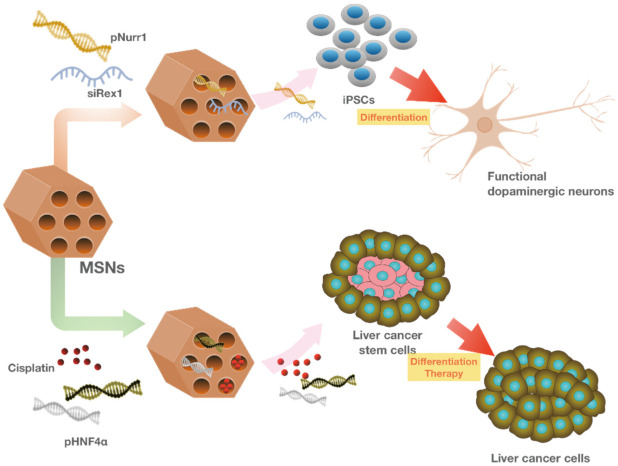
**MSNs as multifunctional nanocarriers in stem cell research.** MSNs are used as potential delivery nanocarriers that can promote the dopaminergic differentiation of induced pluripotent stem cells or drive malignant cancer stem cells to cancer cells with lower tumorigenic potential.

MSNs were also employed for the co-delivery of differentiation factors into pluripotent stem cells (iPSCs) using MSNs. In this strategy, the negatively-charged Nurr1 plasmid DNA (pNurr1) and Rex1 siRNA (siRex1) were absorbed onto the positively charged FITC-conjugated MSNs by electrostatic interactions. Later, the pNurr1-siRex1-FMSN(+) complex was delivered into iPSCs to induce the differentiation of dopamine (DA) neurons. However, the time required for the iPSCs differentiation and the relatively low rate of dopaminergic-positive cells posed limitations on the efficiency of this strategy. Therefore, Chang et al. applied a direct-reprogramming strategy to differentiate fibroblast cells to dopaminergic neurons. In this approach, avidin capped-MSNs were synthesized for the controlled release of the neurogenesis inducer drug, ISX-9 (Isoxazole 9). In addition, three key differentiation genes, Ascl1, Myt1l, and Brn2 were also adsorbed on the avidin-capped MSNs and co-delivered into mouse fibroblasts to direct the differentiation of dopaminergic neuron-like cells [294] (Figure 6 and Figure 7). A recent report suggests the potential application of MSNs for delivering the components required for MSCs mediated myocardial infarction (MI) recovery [285]. In this approach, MicroRNA-21 (miR-21), an important factor for cardiac regeneration [295], was loaded onto an exosome-mimicking complex with MSNs core and delivered into the MI mouse model. This strategy took advantage of the high loading capacity of MSNs and their effective protection against enzymatic degradation. The exosome-mimicking nanomaterial complex was later coated with MSCs membrane to avoid their immune clearance for prolonged circulation time. In addition, MSCs possess antibodies that allow specific targeting to cardiomyocytes.

The search for materials better mimicking the mechanical and adhesion characteristics of extracellular matrix (ECM) has led to the introduction of nanocomposite hydrogels [296]. These smart biomaterials consist of nanoparticles cross-linked with the polymer chain of adhesive hydrogel network via physical or covalent interactions [297]. A great deal of effort has been spent on improving the properties of this system by modifying the synthesis and functionalization of the nanocomposites for clinical translation [298]. However, the efficacy and safety-related issues such as the inflammatory response hampers their translations to clinical practice. It has been reported that MSCs have a positive effect on tissue implantation by infiltrating into the implant and increasing the scaffold integration [299]. In addition, the hydrogel network provides the structural support for MSCs infiltration and proliferation, thereby enhancing the healing process. In addition to the previously described properties of MSNs, it has been shown that they can significantly enhance the mechanical stability and elasticity of the hydrogel [300]. Therefore, along with the conventional application of MSNs as drug delivery vehicles, they can also function as scaffold structures. Fiorini et al. synthesized a novel type of nanocomposite hydrogel with MSNs core and evaluated its efficiency in promoting the MSC infiltration and proliferation [301]. In this design, MSNs were loaded with stromal cell-derived factor-1α (SDF1-1α) to direct stem cells toward the implanted network [302] and covalently bind to the hydrogel. The release of SDF1-1α from the nanocomposite could successfully improve the MSCs migration both in vitro and in vivo. MSNs have also been utilized in nanofiber-based drug delivery to maintain the stem-ness and proliferation of human adipose-derived stem cells (hADSCs). In this system, the curcumin-loaded MSNs were blended with two polymers poly-ε-caprolactone and gelatin via electrospinning. The resulting polymer-based nanofibers CUR-MSNs-NFs could successfully mimic human ECMs demonstrated by a better adhesion of hADSCs and the sustained release of Curcumin leading to a significant increase in hADSCs survival and proliferation [303]. An interesting study exploited MSNs to design stable and biocompatible films that can support human mesenchymal stem cell (hMSC) behavior [289]. In addition, the mesoporous structure of MSNs allows the incorporation of dexamethasone, an inducer of osteogenic differentiation, into MSNs films. In this study, hMSCs were cultured on MSNs films loaded with dexamethasone to support the ECM formation of hMSCs and induce their osteogenic differentiation. The results represent a typical hMSCs morphology, adhesion, and proliferation rates. These findings indicate that MSNs can serve as the coating material with drug delivery ability to guide stem cell behavior.

A very recent study utilized modified platelet lysates (PLMA)-based nanocomposite to induce osteogenic differentiation [193]. PLMA functions as a scaffold for bone regeneration by providing the mechanical stability and bioactive compounds required for cell encapsulation and maintenance [304]. In this study, MSNs were functionalized with calcium and phosphate ions involved in osteoblast differentiation and bone mineral deposition, respectively. The modified MSNs were loaded with dexamethasone (MSNCaPDex) and incorporated in PLMA and hMSCs, resulting in nano-composites hydrogel that can successfully support stem cell adhesion and induce their differentiation toward the osteoblast lineage validated by the stretched morphology and osteogenic differentiation markers. Another similar study also used the MSNCaPDex complex to incorporate into the gelatin methacrylate (GelMA) scaffolds used for the adhesion of hMSCs and the osteogenic differentiation induction [305]. Overall, these findings support the concept of MSN-based system as a delivery system and a scaffold structure for supporting stem cell maintenance and differentiation [306,307].

#### 4.3.2. MSN Application in Cancer Stem Cell Ablation

**Figure 8:**
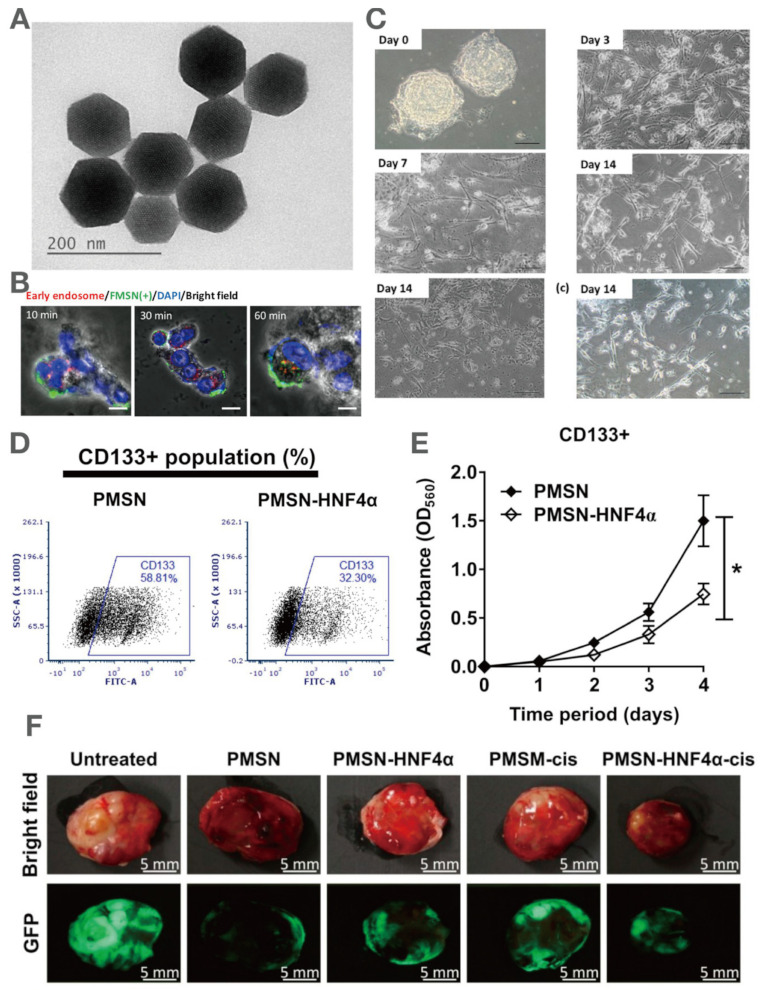
**MSNs as effective nanocarriers for delivering specific genes to promote stem cell differentiation and cancer stem cell ablation.** (**A**) Morphology of MSNs. (**B**) Time-dependent uptake of fluorescent MSNs by induced pluripotent stem cells. (**C**) Generation of functional dopaminergic neurons from induced pluripotent stem cells under pNurr-siRex1-FMSN treatment. (**D**) Flow cytometry indicated the decrease in CD133+ cell population of Huh7 cells treated with PMSN-HNF4α. (**E**) MTT assay showed that PMSN-HNF4α treatment significantly decreased CD133+ cell population of Huh7 cells. * *p* < 0.05 (**F**) Bright field and fluorescent images, showing the synergistic effect of HNF4α and cisplatin co-delivered by PMSN on hepatocellular carcinoma-bearing mice.

The introduction of cancer stem cells (CSCs) has significantly affected the success rate of conventional chemotherapy [308]. Several reports support the idea that CSCs are responsible for cancer relapse, and therefore, the ablation of these cells is critical for cancer therapy [308,309]. A variety of strategies for targeting CSCs have been developed. However, due to CSCs’ unstable surface markers during disease progression and their variations in different patients, these approaches might not be effective [310]. Moreover, cases of cancer cells dedifferentiation to CSCs have been reported [311]. All these imply that standard chemotherapy is no longer a reliable strategy to eradicate cancer cells. Therefore, developing methods that can trigger both CSCs and their differentiated progenies are of great importance [312]. Mandal et al. showed the successful application of MSNs in targeting acute myeloid leukemia (AML) in vitro. In this study, MSNs were functionalized with anti-B220 antibody and loaded with anti-leukemia drug Daunorubicin (DN) for specific targeting of AML stem cells without affecting normal hematopoietic stem cells [313]. In addition, Durfee et al. developed protocell-based nano-carriers with MSNs core for targeted delivery to individual leukemia cells in vivo [314]. In this design, drug-loaded MSNs are encapsulated in a supported lipid bilayer (SLB), which is reported to be responsive to the low pH for controlled drug release. In addition, the surface of SLB can be modified by the conjugation of various ligands according to moiety of the target cell. This strategy combines the advantages of both MSNs and liposomes, boosting the efficacy of the cancer therapy. In another study, polyethyleneimine-modified mesoporous silica nanoparticles (PMSNs) was used to trigger CSCs, associated with the recurrence of hepatocellular carcinoma (HCC) [315]. In this approach, PMSNs were loaded with HNF4α, a key regulator of hepatocyte differentiation to inhibit CSCs proliferation. The delivery of HNF4α-loaded PMSNs into the Huh7 cell line (in vitro model of HCC) could significantly downregulate several stem-ness factors and decrease CSCs proliferation. However, the dual delivery of HNF4α with the traditional chemotherapeutic drug cisplatin showed the most promising outcome in suppressing tumor outgrowth (Figure 7 and Figure 8). Joseph et al. exploited the advantages of MSNs for designing a targeted theragnostic nano vehicle (TTNV) to abolish CSCs using human xenograft murine models [316]. In this approach, antineoplastic doxorubicinand tariquidar were simultaneously loaded onto MSNs to combine the effect of a chemotherapeutic agent and a multidrug-resistant inhibitor. MSNs were functionalized with hyaluronic acid and folic acid to specifically bind with CD44 and folate receptor overexpressed in cancer cells and CSCs, respectively. In addition, MSN pores were capped with pH-sensitive chitosan biopolymers for the controlled drug release. The incorporation of Manganese (Mn) into MSNs increased the biodegradability and excretion of the nano-carriers from the organ. In addition, The Mn-doped MSNs contain Mn–O bonds that are cleaved in response to both redox and mild acidic conditions. This dual drug delivery of MSNs offers an efficient strategy to maximize the targeting delivery of therapeutics on demand. Another study by Liu et al. also utilized MSNs to target CSCs both in vitro and in vivo models [317]. The aim of this study was to minimize the side effects of chemotherapy by downregulating cyclooxygenase-2/prostaglandin-E2 (COX-2/PGE2) signaling known to contribute to the cancer relapse. MSNs were loaded with the doxorubicin and functionalized with poly(β-cyclodextrin) as the gatekeeper to restrict the premature delivery of the chemodrug. In this advanced design, celecoxib, a redox-responsive COX-2 inhibitor, was employed as a structural element to connect MSNs’ surface with PCD gatekeeper via disulfide bonds. Therefore, exposure to the reducing microenvironment resulted in the cleavage of this disulfide bond and the co-release of celecoxib and doxorubicin into tumor. Li et al. developed a magnetic MSN-based drug delivery system that is able to release the drug into the nucleus of CSCs in vitro [318]. This nano-system possess a core-sell structure including magnetic iron-oxide-cores which can generate the heat under alternating magnetic field (AMF). The MSN pores are loaded with tirapazamine (TPZ), with a selective toxicity against hypoxic CSCs. MSNs surfaces were functionalized with the conjugation of CD133 antibody with temperature-responsive polymer and nucleus targeting TAT peptides. After the targeted delivery of MSNs-TPZ into CSCs, AMF was used as an external stimulus to break the temperature-sensitive bonds, resulting in the exposure of TAT peptide for the release of the TPZ drug into CSCs nucleus.

#### 4.3.3. MSN Application in Stem Cell Labeling

As previously mentioned, MSNs have been functionalized with diverse imaging agents, such as Gd^3+^ and ^64^Cu, to integrate diagnostic and therapeutic applications. Therefore, MSNs with imagining ability can be utilized for stem cell tracking [269]. The in vivo tracking of transplanted stem cells allows a guided and more precise therapy. Rosenbrand et al. showed that modifications of MSNs with lipids and PEG can improve their ability for stem-cell tracking by increasing the labeling efficiency and signal intensity [319]. Kempen et al. synthesized MSNs to serve as both drug carriers and labeling agents for ultrasound and MRI [320]. As described in Section 4.2, the ultrasound combines the image-guided delivery and quantitation abilities, while MRI provides excellent image resolution for diagnosis. This theragnostic nano-carrier could detect up to nearly 9000 MSCs with no reported cytotoxicity after an in vivo implantation. In addition, no trace of these nano-carriers was detected after about three weeks, indicating their desirable biodegradation property. Huang et al. used MSNs to develop a multifunctional nano-carrier with targeted cancer delivery and labeling properties. In this approach, modified MSNs (HA-MSNs) with multi-modality optical, PET, and MRI abilities were utilized to monitor mesenchymal stem cells. Their potential in vivo applications were further confirmed by successful targeted delivery to the orthotopic glioblastoma xenograft model [321]. Chen et al. developed a cup-shaped structure of MSNs for ultrasound imaging of mesenchymal stem cells with negligible toxicity in vivo. This structure improved the ultrasound contrast of stem cells due to its positive charge as well as inherently increased echogenicity [322].

MRI has been employed to noninvasively track stem cell distribution and the fate of grafted stem cells [323]. To monitor stem cell distribution, cells must be labeled magnetically via endocytic internalization, but this technique is weighed down by several shortcomings. For example, the high concentration of magnetic particles or their long incubation time with cells causes low intracellular labeling efficacy [324,325]. Thus, developing a method to improve cellular internalization is in great demand. As mentioned earlier, silica (SiO_2_) and, in particular, MSNs, serve as biocompatible nanomaterials with surfaces that are easy to be modified for bio-conjugation [326,327]. Liu et al. synthesized silica-coated core-shell superparamagnetic iron oxide (SPIO) nanoparticles for magnetic labeling and detection of hMSCs in vitro and in vivo [328]. Later, they conjugated the non-fluorescent silica core-shell SPIO (SPIO@SiO2) with FITC incorporated MSNs (FITC-MSNs), named as Mag-Dye@MSNs, and reported their safety and efficiency for HMSCs labeling, as demonstrated by the MRI analyzer. Notably, no adverse effects on cell growth, viability, or differentiation was observed. In addition, they verified the importance of MSNs in this NP design by comparing the MRI signal strengths of hMSCs labeled in the presence or absence of MSNs, manifested by a higher labeling efficiency of Mag-Dye@MSNs versus SPIO@SiO2. Hsiao et al. also used Gd-based chelates MR contrast agent with T1-enhancing properties for monitoring hMSCs by clinical 1.5-T MRI imaging system [329]. In this approach, dual functional Gd-fluorescein isothiocyanate MSNs (Gd-Dye@MSN) with green fluorescence and paramagnetism could efficiently label MSCs for MRI detection without affecting their viability, proliferation, and differentiation abilities.

### 4.4. MSN Application in Anti-Cancer/Tumor Therapy

The application of MSNs for various cancer therapies has received much attention over the past few years [330,331,332,333]. It provides a comprehensive approach for diagnosing, preventing, and treating this complicated and multistep disease that affects numerous cellular processes. For any anti-cancer therapy, the goal is to efficiently destroy cancerous cells with minimal damage to normal cells. Although most targeted drug delivery systems suffer from premature drug release before reaching target sites, tremendous efforts have been made to achieve tumor-specific delivery and controlled release of anticancer agents [157,173,334,335]. As mentioned in Section 6 to gain control over releasing drugs, stimuli such as pH [13,14,165,170], redox potential [163,174,175,336,337], enzymes [166], light [164], magnetic field [12], and ultrasound [338] have been used.

Nonetheless, regardless of the type of anti-cancer therapy, one of the biggest challenges in the delivery of hydrophobic drugs is their low solubility in aqueous media, which hampers their administration through the intravenous route. In light of this, MSNs have been shown as favorable nanocarriers for drug targeting applications in anti-cancer treatment [339,340]. For example, drugs such as camptothecin (CPT) [340] and paxlitaxel [341] show excellent anti-cancer ability in vitro but have low success in vivo due to their limited solubility. However, owing to the customizable pore size and shapes of MSNs, drugs can be loaded on the surface of MSNs, which significantly increase their solubility. Additionally, the engineered properties of multifunctional MSNs allow the development of new strategies for controlled release. For example, Bhavsar et al. synthesized DOX-MSN-SS-CH-FA to actively target breast cancer cells in vitro. In this design, MSN pores were loaded with the anti-cancer drug doxorubicin (DOX) and capped with chitosan and folate gatekeepers, responsive to redox and pH stimuli, respectively. DOX is released from MSNs upon exposure to acidic-redox conditions (pH 5.5, 10 Mm GSH) representative of the cancer environment. In addition, they reported that this MSN-based design is safe for I.V. administration with increased biocompatibility [342]. The most desirable drug delivery system should possess high drug-loading efficiency, site-specificity, and the capacity of controlled drug release to achieve effective and precise therapies. Different MSN modifications have been explored to generate various single- and multi-modal anti-cancer therapeutics [278,343,344]. In this section, we highlight some recent advances in the development of MSN-based anti-cancer therapies, such as photodynamic therapy (PDT), sonodynamic therapy, chemotherapy, radiotherapy, immunotherapy, and gene therapy.

#### 4.4.1. MSN Applications in Photodynamic Therapy (PDT) and Sonodynamic Therapy (SDT)

**Figure 9:**
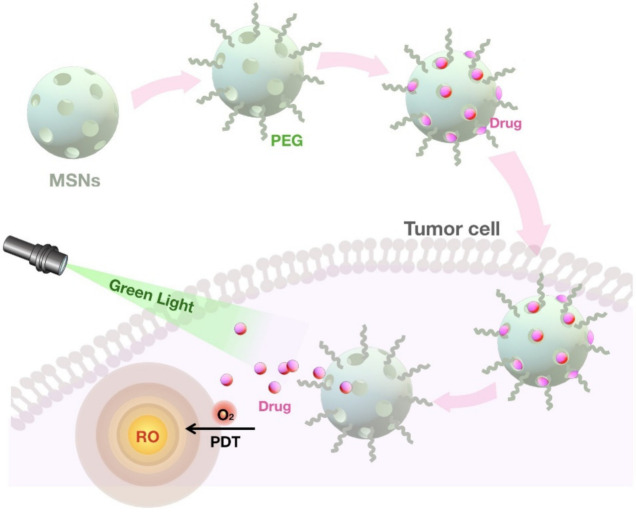
**MSNs in the application of anti-cancer PDT.** PEG-modified MSNs deliver photosensitizers into tumor cells and trigger their release upon the irradiation of green light, leading to the generation of reactive oxygen and cancer ablation.

Photodynamic therapy (PDT) is an established method for cancer treatment, which utilizes a combination of non-toxic light, photosensitizer (PS), and oxygen [24]. In this approach, when the PS, (used as a drug) is exposed to a specific wavelength of light, it generates reactive oxygen species (ROS), including single oxygen and free radicals, that would kill the nearby cells. However, since PS molecules do not exhibit cytotoxicity until triggered by light at a specific site, PDT results in much less collateral damage than conventional anti-cancer therapy methods. Despite this advantage, PDT agents also have their own limitations. For example, PSs are hydrophobic and may cause altered oxygen quantum fields, which makes their deliveries challenging. Besides, the poor biodistribution of PDT agents in organs such as skin can cause increased accumulation and skin sensitivity [345]. Furthermore, light cannot penetrate tissue beyond 3 mm due to the high tissue absorption [346]. In addition, PDT agents show low tumor specificity compared with normal tissues [345]. Over the years, MSNs have been found as a potential drug delivery platform for PDT due to their controllable synthesis, loading, diverse surface functionalization, and biocompatibility [347,348,349]. For example, Kuang et al. developed an MSN system (MSN-PEG@Cur) that enhances the bioavailability and anti-cancer effect of the photosensitizer curcumin (Cu). MSNs were first fabricated with polyethylene glycol (PEG) for the easier escape from phagocytosis and then Cur were loaded in MSN pores. After the cellular uptake, curcumin can be released from MSN-PEG@Cur to induce anti-cancer effect in human cervical cancer (Hela) cells [147] (Figure 9).

In another study, Yang et al. showed the potential application of the rod-shaped MSNR@MoS2-HSA/Ce6 nanocomposites in both PDT and photothermal therapy. In the other study, rod-shape MSNR@MoS2-HSA/Ce6 nanocomposites were synthesized for photothermal therapy and PDT. In this advanced design, MSNs were coated with (MoS2) Graphene-like 2D nanomaterial molybdenum disulfide (MoS2) as a photothermal agent, resulting in MSNR@MoS2 nanocomposite with photothermal therapy ability. This is followed by the chemical conjugation of chlorin e6 (Ce6) with florescent property and PS to endow the nanocomposite (MSNR@MoS_2_/Ce6) with PDT ability upon NIR irradiation. This nanocomposite was functionalized by human serum albumin (HSA), which specifically binds with the secreted protein acidic and rich in cysteine (SPARC) overexpressed in many tumor cells. They demonstrated that these MSNR@MoS_2_-HSA/Ce6 exhibit superior tumor cellular uptake and enhanced Ce6 release under NIR laser irradiation, demonstrating a promising alternative for inhibiting tumor growth by the combination of PDT and photothermal therapy [350].

In another approach, Zhan et al. fabricated a biocompatible superparamagnetic Fe_3_O_4_-MSN, conjugated with a PEG-b-poly(aspartic) polymer for the magnetic and pH-triggered delivery of Rose Bengal (RB) as the PS to tumor sites. Compared to free RB, the MSN-mediated delivery of RB allowed more effective endocytosis by mouse melanoma B16 cells, which resulted in enhanced cellular uptake. Furthermore, the improved therapeutic specificity demonstrated by the specific tumor targeting and the reduced tumor size in in vivo tumor-bearing mice as well as the decreased cellular toxicity, suggested the potential application of MSNs in improving the efficiency of clinical PDT [348].

An important limitation implicit in the assessment of PDT efficacy in clinical practice is the insufficient generation of ROS. To address this shortcoming, Shen et al. combined PS with DC50, responsible for a significant reduction in the speed of ROS consumption [351]. To enhance the light-triggered ROS accumulation in cancerous cells, inhibition of copper transfer by DC50 (C_17_H_14_BrF_2_N_3_OS) is commonly used to reduce ROS scavenging. In this approach, a light-responsive MSN-based platform was developed by loading Au nanoparticles on the surface of mesoporous silica-coated UCNPs resulting in the generation of UPSD@Au nanocomposite. In this design, MSNs were loaded with DC50 and a PS (silicon phthalocyanine dihydroxide, SPCD) to increase the ROS production and trigger PDT upon light exposure, respectively. This MSN-based platform also allows the controlled release of dc50 in response to thermal stimuli generated by the photothermal transfer process from UCNPs to Au NPs upon near-infrared laser irradiation. Meanwhile, SPCD converts near-infrared light (NIR) into visible light which leads to the activation of SPCD, and helps the penetration of deep lesions upon light excitation. Overall, this MSN-based platform contributes to more efficient PDT in both in vitro and in vivo studies.

To further enhance ROS-mediated anti-cancer treatment, the synergistic effect of PDT and chemodynamic therapy (CDT) has been explored by several research groups [278,344,352]. For example, Zhu et al. synthesized a composite core–shell-structured nanozyme (MS-ICG@MnO_2_@PEG) that consists of MSN core and a MnO_2_ shell loaded with the PS indocyanine green (ICG) [353]. This nanocomposite was coated with PEG and employed as a PDT/CDT therapeutic agent for the ROS-mediated anti-cancer treatment in 4T1 breast tumor cells and tumor-bearing mice. These nanozymes catalyzed H_2_O_2_ to produce O_2_ for enhanced PDT and simultaneously consumed GSH to generate –*OH for enhanced CDT. In in vivo studies, these MS-ICG@MnO_2_@PEG nanozymes selectively accumulated at tumor sites and inhibited tumor growth and metastasis. Together, these findings suggested MS-ICG@MnO_2_@PEG nanozymes as an effective platform for ROS-mediated anti-cancer treatment by enhancing the combination of PDT and CDT.

In another study, the synergistic anti-tumor effect of PDT and CDT has also been achieved with biodegradable pH-responsive HMSNs. This nanomaterial comprises the amino-abundant chitosan (CS) anchored on the surface of HMSNs, Glycidoxypropyl-tri-methoxy-silane (GPTMS) as a crosslinker to anchor CS, and folic acid (FA) used to improve the targeting abilities (HMSN-GM-CS-FA) [88]. These HMSNs were applied in the dual delivery of a PS pheophorbide a (PA) and the chemodrug doxorubicin exhibiting excellent encapsulation capacities with controlled particle size and improved uptake efficiency. These results suggest the efficiency of MSNs as dual delivery systems for combined PDT and CDT in clinical applications. In a similar study, the PS chlorin e6 (Ce6)-loaded MSNs generated ROS upon laser irradiation to trigger the controlled release of doxorubicin and doxycycline, which further enhanced ROS production and showed promising outcomes in treating osteosarcoma [354]. This MSN-based amplification of ROS generation induces long-term overaccumulation of oxidative stress in PDT, resulting in the increased sensitivity of osteosarcoma to chemotherapy.

Conventional prostate treatment strategies are unable to eradicate prostate cancer effectively, especially for castration-resistant prostate cancer. Xu et al. developed an MSN-based nanotherapy platform by combining photothermal and PDT via putative photothermal conversion by gold nanorods (responsible for the light-to-heat energy transfer) and free radical generation by water-soluble radical initiator 2,2′-azobis[2-(2-imidazolin-2-yl)propane] dihydrochloride [146]. The bombesin peptide was conjugated onto the coating layer of MSNs for the targeted delivery to prostate cancer cells. In the castration-resistant prostate cancer cells, this MSN-based nanotherapy platform showed a remarkable efficacy of photothermal therapy and enhanced thermodynamic therapy with the generation of free radicals and the upregulation of phosphorylated forms of p38 and JNK proteins, two key mediators involved in cellular stress responses. In addition to its prominent eradication of prostate cancer, this MSN-based nanotherapy platform showed excellent biocompatibility. These findings suggested that the combination of photothermal, thermodynamic, and site-specific drug delivery using the MSN-based nanotherapy platform can serve as a novel approach for the treatment of castration-resistant prostate cancer.

Polymerized hollow mesoporous organosilica nanoparticles (HMONs), a derivative of MSNs, have also shown promising results in anti-tumor therapy. Li et al. combined the synergistic effect of PDT/CDT for improving ROS-mediated pancreatic ductal adenocarcinoma therapy [86]. In this design, ultrasmall gold nanoparticles were immobilized on the hollow cavity of HMONs, which enables the efficient conversion of glucose into H_2_O_2_ required for CDT. Later, the deposition of Cu^2+^-tannic acid complexes on the surface of HMONs (HMON-Au@Cu-TA) converts the generated H_2_O_2_ to the free radical OH, providing the supply for the tumor cell ablation. In addition, collagenase was loaded into HMON-Au@Cu-TA to degrade the collagen I fiber and enhance the penetration of HMONs and O_2_ infiltration. Collectively, the innovative design of HMONs offers a novel alternative for enhancing ROS-mediated antitumor therapy.

Another study fabricated a novel MSN-based nanocomposite in which photoluminescence molecules perylene diimide were hybridized within the cavity of HMONs (HMPDINs). These HMPDINs were able to amplify the fluorescence and photoacoustic signals, making them ideal candidates for enhanced fluorescence and photoacoustic imaging. In this design, the organosilica shell was chelated with radioactive isotope ^64^Cu for PET imaging system. The controlled drug release was achieved by the introduction of a thermos-responsive polymer into the cavity of HMPDINs. The release of the drug was triggered by the deformation of the polymer in response to the heat generated from near-infrared laser irradiation. This novel nanocomposite with organic–inorganic properties improves the cancer theragnostic efficacy and may open a new avenue for designing advanced theragnostic platforms [355]. Furthermore, MSNs have also been used to co-encapsulate agents that might interfere with each other. For example, the GOx-MSN@MnPc-LP nanoparticles have been designed to co-deliver hydrophilic enzymes and hydrophobic photosensitizers [356]. These MSNs are simultaneously loaded with the hydrophilic glucose oxidase (GOx) and the hydrophobic manganese phthaleincyanide (MnPc) in different compartments, demonstrating the combinatory effects of different agents in inhibiting tumor growth in spatially controlled PDT.

The development of PDT showed remarkable progress in anti-cancer therapy. However, the traditional PDT or photothermal therapy is usually hindered by the low penetration depth of tissues. The sonodynamic therapy (SDT) was developed from PDT using low-frequency ultrasound and has received increasing attention as a new non-invasive treatment. Compared with PDT, SDT can penetrate deeper tissue due to its non-radiative emission and low tissue attenuation coefficient. SDT has shown great potential to remove proliferative scars, kill pathogenic microorganisms, as well as remarkable efficacy in treating solid tumors, leukemia, and atherosclerosis [357,358,359]. Considerable effort has been made to explore MSN applications in synthesizing more effective sonosensitizers, for example, the TiO_2_ MSNs chelated with heavy metals Au, Pt, or Ag [360,361,362]. Another method commonly used to produce sufficient ROS for tumor growth suppression is the cavitation-inducible sono-theragnostic MSNs. For example, Lee et al. developed the PFH@PEGylated mesoporous silica–titania nanoparticles (P-MSTN), a gas precursor perfluorocarbon-loaded nano-photosensitizer. They demonstrated that PFH@P-MSTN could enhance ROS generation to induce tumor cell death in response to ultrasound exposure. Following systemic administration in tumor-bearing mice, the PFH@P-MSTN tended to accumulate in the tumor site through the passive targeting mechanism [363].

Zuo et al. synthesized a biocompatible Cu_2−x_S-RB@DMSNs-AE105 carrier to overcome the shortcoming attributed to the conventional therapeutic strategies employed for the oral squamous cell carcinoma (OSCC) treatment [364]. In this design, dendritic mesoporous silica nanoparticles (DMSNs) were conjugated with the AE105 for the targeted delivery to OSCC cells with overexpressed urokinase plasminogen activator receptor (uPAR) on their membrane. The photonic active ultrasmall Cu_2−*x*_S NPs and sonosensitizer Rose Bengal were constructed and loaded onto DMSNs without affecting the three-dimensional architecture of nanoparticles. Subsequently, the NIR laser and ultrasound irradiation enabled the respective in situ photonic-hyperthermal and sonodynamic effects to induce the death of OSCC. These DMSNs have demonstrated their potential in the efficient eradication of tumor cells via a synergetic dual-therapy modality.

One of the challenges of ROS-based therapies is the high concentration of glutathione (GSH) within tumor cells, which antagonizes ROS and thereby reducing the treatment efficiency. To address this issue, Huang et al. developed a platelet membrane coated nanosystem (PSCI) with MSN core to encapsulate the cinnamaldehyde (CA) as the oxidative stress amplifier and the sonosensitizer IR780 (MSN-CA-IR780) for amplifying the effect of SDT. The platelet membranes, as the outer layer, could stabilize the nanacarriers in the blood by prolonging their circulations. In addition, both CA and IR780 contribute to the tumor ablation by generating ROS and suppressing GSH in the tumor microenvironment, respectively [365].

#### 4.4.2. MSN Application in Chemotherapy

Chemotherapy is one of the most established and important anti-tumor treatments to date. However, the effectiveness of traditional chemotherapy is limited by its poor selectivity, poor aqueous solubility, high systemic toxicity, and multidrug resistance [91,366,367]. MSNs have emerged as a potential drug delivery system that can both improve chemotherapy on its own [368,369,370] as well as in combination with other cancer therapies [90,371,372]. One of the major challenges of chemotherapy is the multidrug resistance (MDR) that is commonly attributed to the overexpression of the transmembrane efflux pump P-glycoprotein (P-gp) in cancer cells. To suppress such drug efflux, MSNs have been used to simultaneously co-deliver anti-cancer drugs and P-gp inhibitors to the tumor tissues [373]. These MSNs were loaded with the doxorubicin, coated with an extracellular-tumor-acidity-responsive polymer shell (PEG-b-PLLDA) to prolong the blood circulation time of these nanocarriers, and cationic β-cyclodextrin-PEI (CD-PEI) gatekeepers that allow the release of doxorubicin in response to the intracellular acidity and glutathione within the cancer cell environment. These nanocarriers were shown to significantly suppress MDR tumor growth without any systemic toxicity in tumor-bearing mice, demonstrating their potential applications for clinical translation.

Erlotinib is a potent targeted drug to treat non-small cell lung cancer (NSCLC) for oral administration. However, poor solubility, low oral bioavailability, and capricious toxicity of this drug hinder its clinical applications. Zhou et al. developed an injectable matrix (ERT@HMSNs/gel) using HMSNs and thermosensitive hydrogel to encapsulate and allow the sustained release of Erlotinib [89]. This injectable matrix is a flowing solution at room temperature while transforms into a non-flowing gel structure under physiological temperature. Besides, this ERT@HMSNs/gel matrix allowed long-term retention of the drug in vivo along with an optimal balance between anti-tumor effect and biosafety in a mouse NSCLC xenograft model. Collectively, this ERT@HMSNs/gel matrix may serve as a promising drug delivery system for the treatment of NSCLC.

One of the most widely explored MSN-based combinational chemotherapy is the integration of phototherapeutics and chemotherapeutics [374,375,376,377,378,379]. Fang et al. constructed dual-stimuli responsive 5-fluorouracil (5-FU)-loaded and indocyanine green (ICG)-conjugated mesoporous silica-coated gold nanorods (GNR@SiO_2_-5-FU-ICG) for the multimodal imaging-guided synergistic therapy [277]. ICG conjugated onto the surface of nanocarriers was exploited for fluorescence imaging and PDT. In addition, 5-FU was loaded into the nanocarriers for the chemotherapy application. Under the acidic tumor microenvironment, the protonation of surface silanols enervated the electrostatic interaction between 5-FU and silica shell. Moreover, the laser-induced heat dissociated the electrostatic interaction between 5-FU and silica shell. Both the intracellular acidity and the NIR irradiation could trigger the release of 5-FU from the GNR@SiO_2_-5-FU-ICG nanocarriers. The NIR irradiation resulted in the generation of singlet oxygen and heat for PDT and photothermal therapy, respectively. These GNR@SiO_2_-5-FU-ICG platforms hold great promises in trimodal synergistic therapy, including chemotherapy, PDT, and photothermal therapy under the guidance of multimodal imaging. Importantly, with the guided multimodal imaging, the treatment efficacy was remarkably improved in xenograft tumor-bearing mice. This study provided a new concept that integrates multiple diagnostic and therapeutic modalities into a single platform, raising hopes for a personalized nanomedicine-based approach with both diagnosis and treatment approaches [277].

Wu et al. developed a high-resolution Förster resonance energy transfer (FRET-based two-photon MSNs (MTP-MSNs) for single-excitation multiplexed intracellular imaging and targeted cancer therapy [380]. This MSN-based nanocarrier comprises two major parts, including the multicolor two-photon mesoporous (MTP)-MSNs and the cancer-targeting aptamers, that also serve as gatekeepers for the controlled drug release. The aptamer-capped MTP-MSNs nanocarriers were loaded with doxorubicin and specifically internalized into cancer cells, followed by the cap opening and subsequent drug release into the tumor environment. Furthermore, the MTP-MSNs could serve as ideal contrast agents, providing two-photon multicolor fluorescence with improved spatial tissue localization and increased image depth. In summary, these MTP-MSNs using two-photon multicolor fluorescence offered both a traceable therapeutic strategy for targeted cancer delivery and multiplex intracellular imaging. In another study, Dong et al. fabricated Janus nanoplatforms that integrated chemotherapy/radiotherapy/photothermal therapies for treating liver cancer. Both in vitro and in vivo studies indicated the safe and effective anti-tumor activities of these Janus nanoplatforms [381,382,383]. In addition, the Janus-structured MSNs could further act as CT-imaging agents for the diagnosis of liver cancer. This Janus-structured MSN-derived nanoplatform provided novel theragnostic strategies for unresectable hepatocellular carcinoma.

Immunogenic chemotherapy, another type of chemotherapy, has been known to induce immunogenic cancer cell death using certain chemo-drugs. Although immunogenic chemotherapy can induce T cell anti-tumor immunity, it also contributes to the upregulation of immunosuppressor indoleamine-2,3-dioxygenase (IDO) [384,385]. Interestingly, MSNs have also been used to co-deliver the chemo-drug doxorubicin and IDO inhibitor, 1-methyl-d-tryptophan (1-MT), in immunogenic chemotherapies. MSNs could specifically deliver 1-MT into the tumor extracellular compartment and doxorubicin into the intracellular endosomal compartment, respectively. The release of 1-MT effectively suppressed IDO activity and its immunosuppressive effect, while the release of doxorubicin led to the immunogenic cancer cell death, accompanied by the effector T cell infiltration [386]. In a colon tumor xenograft mouse model, this doxorubicin- and 1-MT-loaded MSNs amplified both tumor local and systemic antitumor immunity, leading to the suppression of primary tumor growth and extending recipient survival.

In another study, Liu et al. used MSNs coated with lipid bilayers (silicasome) to deliver activated platinum chemo agent used for immunogenic chemotherapy in a pancreatic ductal adenocarcinoma (PDAC) preclinical model [387,388]. The silicasome coating improved the colloidal stability of this nanocarrier after the intravenous injection. MSNs encapsulating the activated platinum could significantly enhance the intratumor drug delivery and immunogenic cancer cell death with minimal bone marrow toxicity. The lipid bilayer-coated MSNs holds great promise in platinum-based chemotherapy drugs.

#### 4.4.3. MSN Application in Radiation Therapy

Targeted alpha radiation employs radioactive agents with short-range and high linear energy for the delivery of highly cytotoxic doses to the tumor site with minimal damage to surrounding healthy tissues. This in-development approach has emerged as a promising strategy for highly targeted cancer treatment [389,390]. Several radionuclides (including ^211^At, ^213^Bi, and ^212^Pb) and isotopes (such as ^225^Ac) with single-particle and multiple-particle emissions have been clinically investigated as candidates for targeted alpha therapy [391,392,393,394].

However, these metal complexes were reported to cause cytotoxicity due to their chemical properties such as the formation kinetics, stability, and coordination chemistry. MSNs, on the other hand, have been explored for their targeting capabilities MSNs and biocompatibility as a radiotherapy agent [239,395,396,397,398]. For example, Pallares et al. constructed the radioisotope-labeled MSNs (^225^Ac-HOPO-MSN and ^238^Pu-HOPO-MSN) via the conjugation of nanoparticles with transferrin to promote nanoparticle accumulation at tumor sites and a chelator with high specificity and affinity to bind with f-block elements. The resulting radioisotope-labeled MSNs exhibited specific targeting delivery, enhanced cytotoxic effect and clearance in vitro, as well as negligible deposition in bones in vivo. These results highlighted the potential of radioisotope-labeled multifunctional MSNs as a novel nanotherapeutic agent for targeted alpha therapy [399]. In another study, Li et al. examined the anti-cancer effect of hydroxychloroquine-loaded HMSNs in vitro and in vivo. Comparing with the free hydroxychloroquine, HMSN-mediated delivery of hydroxychloroquine largely increased the intracellular uptake of hydroxychloroquine, leading to effective inhibition of radiation-induced cytoprotective autophagy in cultured colon cancer cells. In a mouse tumor xenograft model, hydroxychloroquine-loaded HMSNs tended to accumulate in tumor tissues, resulting in a significant decrease in radio-resistance and improved radiosensitivity by the inhibition of autophagy [73]. Besides enhancing radiosensitivity in radiotherapies, addressing the safety issues associated with radiation exposure has remained a challenge. In this regard, MSNs have been used to provide a targeted treatment platform by minimizing radiation exposure to non-cancerous cells. For example, Hargrove et al. synthesized and loaded MSNs with a stable isotope of Holmium-165 [400]. These Holmium-165-loaded MSNs were placed in a thermal neutron flux that converts Holmium-165 to its radioactive form, Holmium-166. Holmium-166 can emit high-energy β-particles and γ- photon, making this radioactive isotope a useful tool for cancer treatment and single-photon emission computed tomography. The relatively short half-life of Holmium-166 helps with decreased radiation exposure to the patient after administration. These particles tend to accumulate within the tumor tissues selectively and passively, therefore, limiting the exposure of healthy tissues to radiation.

Remarkable efforts have been made for developing MSNs-assisted combinational anti-tumor therapy, such as the combination of radiotherapy and PDT, the X-ray-induced PDT, as a PDT derivative. The new derivative approach has demonstrated better anti-cancer effects than radiotherapy alone. Hence, this therapeutic approach is considered as the combination of PDT and radiotherapy, rather than PDT alone [401]. Ahmad et al. enriched scintillation of CeF_3_ nanoparticles through co-doping with Tb^3+^ and Gd^3+^ (CeF_3_:Gd^3+^, Tb^3+^). Next, these co-doped nanoparticles were coated with MSNs and further loaded with Rose Bengal. The resulting nanocarriers could be used for CT- and MRI-guided synergistic radiotherapy and PDT upon the irradiation of X-rays [402]. Comparing with single radiotherapy, the X-ray-stimulated synergistic radiotherapy and PDT induced more efficient tumor regression in tumor-bearing mice. The efficient tumor regression after the X-ray-stimulated synergistic radiotherapy and PDT was achieved by the starvation of non-essential amino acids involved in protein and DNA synthesis as well as metabolic pathways promoting growth and disease progression. Overall, these findings highlight the utility of MSN-based modern scintillating radio-enhancer nanoparticles for the treatment and diagnosis of tumors within deep tissues. In another study, Sun et al. fabricated an MSN-derived nanosensitizer, which is comprised of the PS Rose Bengal, the silicates with Zn-Mn-dopants (ZSM), and the cyclic arginylglycylaspartic acid (RGD) peptide that can specifically bind to integrin receptor overexpressed in tumor cells [403]. This MSN-based RB-ZSM-RGD nanosensitizers were able to effectively accumulate in tumor sites and suppress tumor progression upon low-dose X-ray irradiation with minimal side effects on normal tissues. These observations imply the potential of MSN-based nanosensitizers to overcome the existing limitations of PDT and radiotherapy, thereby offering an alternative approach for targeted cancer therapy.

#### 4.4.4. MSN Application in Gene Therapy

MSNs have also been applied in delivering nucleic acid therapeutics to specific cells to silence or inhibit the expression of certain proteins involved in cancer development [110,404,405]. Nucleic acids usually cannot cross cell membranes without first being destroyed by DNAases or RNAases in the biological environment. In addition, they are highly specific with their subcellular localization. For example, plasmid DNA needs to reach the cell nucleus while interfering RNA (such as short interfering RNA or siRNA) has to be delivered into the cell cytoplasm [406]. Therefore, MSNs can be ideal targets for encapsulating different genes in their porous structure and enter the cells via endocytosis [407]. Notably, many of the MSNs synthesized for gene therapy are multi-functional and involved in different therapeutic applications. For example, Du et al. constructed the magnetic MSNs-loaded lipid microbubbles for gene transfection and ultrasound-mediated imaging in ovarian carcinoma cells. In this approach, plasmid DNA was encapsulated in MSN pores and protected from the nuclease degradation by modification of MSNs through grafting of PEI. Later, the plasmid DNA-carrying magnetic MSNs were loaded in the lipid microbubbles [408].

In response to the magnetic field, magnetic MSNs loading lipid microbubbles were attracted to the tumor sites. The ultrasound triggered the destruction of the microbubble, the release, and ultimately the efficient delivery of magnetic MSNs to the tumor site. These processes were achieved mainly by the ability of MSNs to increase penetrating the blood-tumor barrier as well as enhancing the tumor cell membrane permeability [408]. Asparagine is a key regulator of amino acid homeostasis, anabolic metabolism, and cell proliferation in cancer cells. The sleeping beauty plasmids allow the stable integration of the genes into the genome of target cells and mediate their sustained expressions. Chang et al. developed amine-functionalized MSNs to co-deliver the asparaginase and sleeping beauty plasmids, thereby integrating the asparaginase gene into the genome of human lung adenocarcinoma cells [409]. The intracellular expression of asparaginase induced cytotoxicity, and the combination of chemotherapy and the asparaginase gene therapy further enhanced this cytotoxicity. This work indicated the excellent potential of MSNs for the delivery of therapeutic genes into cancer cells. Tsai et al. used MSNs to co-deliver conventional chemotherapeutic drug cisplatin and the master regulator of liver-specific gene expression, HNF4α, to simultaneously undergo chemotherapy and gene therapy in hepatocellular carcinoma. This combinational therapy successfully dedifferentiated hepatocellular carcinoma cells with cancer stem-like properties into regular liver cancer cells, exhibiting poorer tumorigenic potential. This research suggested the application of MSNs as an efficient carrier for combining chemo-gene therapies to treat malignant cancer cells [315].

NF-κB signaling has been associated with various tumors, and direct blocking of the NF-κB p65/p50 Rel proteins can serve as a potential target for anti-cancer therapies. Chen et al. successfully utilized an MSN/antibody complex to target NF-κB and catch the active NF-κB p65 subunit using p65 specific in the perinuclear region in head and neck squamous cell carcinoma. A cell-penetrating peptide TAT was conjugated onto MSNs to mediate the non-endocytosis cell-membrane transducing and converge toward the perinuclear region. The binding of MSNs/p65 antibody to NF-κB p65 subunit significantly increased its size, which in turn prevented the translocation of p65 into the nucleus, thereby blocking the subsequent cancer-associated gene expression. This antibody therapy, targeting transcription factors with “size exclusion blocking” can be used as a novel approach for the treatment of various cancers [155].

#### 4.4.5. MSN Application in Immunotherapy

Cancer immunotherapy has emerged as a powerful treatment strategy to prevent the recurrence of cancer owing to its high antigen-specificity and immune memory [410,411,412,413]. The key to a successful antigen-adaptive immune response is to develop an efficient antigen delivery system that improves the activation of T cells and dendritic cells. Given that MSNs can efficiently load high amounts of cargo, it is feasible to use MSNs for the delivery of cancer vaccines comprising protein antigens and immune adjuvants [414,415]. For example, Lee et al. developed an antigen delivery system for enhancing the activation and maturation of dendritic cells using HMSNs with extra-large mesopores (H-XL-MSNs) and a core composed of iron oxide nanoparticles [85]. The surface of HMSNs was coated with poly(ethyleneimine) to change the surface charge of MSNs, allowing the efficient loading and release of antigens. Delivering the cancer vaccine using these H-XL-MSNs effectively enhanced both the activation of dendritic cells and antigen-specific cytotoxic T cells in vitro. Furthermore, the in vivo studies in tumor-bearing mice showed a significant improvement in tumor suppression and recipient survival. These results suggest that HMSNs with extra-large pores are excellent carriers for cancer vaccines [85]. Besides, cyclic diguanylate monophosphate can activate the stimulator of interferon genes (STING) pathway, leading to the enhanced tumor immunogenicity, which in turn reverses the immunosuppressive tumor microenvironment. Therefore, Chen et al. synthesized the RITC fluorescent MSNs modified with poly(ethylene glycol) and loaded with an ammonium-based cationic molecule (TA) with negatively charged cdG, forming the nanocomposites cdG@RMSN-PEG-TA [416]. In cultured macrophages in an in vitro breast tumor microenvironment, the treatment of cdG@RMSN-PEG-TA induced the phosphorylation of STING (Ser365) and secretion of various cytokines. FcdG@RMSN-PEG-TA treatment enhanced infiltration of CD4+T, CD8+T, and other leukocytes in the tumor microenvironment in breast tumor-bearing mice. This study suggested novel immunotherapy using an MSN-based delivery of STING agonist for cancer therapies. Wagner et al. fabricated MSNs with a pH-responsive delivery system of the synthetic immune response modifier R848, a Toll-like receptor 7 and 8 agonist, for anti-cancer immunotherapy [417]. MSNs loaded with the synthetic immune response modifier R848 could be rapidly taken up by antigen-presenting cells into the acidic environment of the lysosome and activate the immune and dendritic cells. These findings indicate that MSNs are efficient nanocarriers for the delivery of cancer vaccines.

Checkpoint blockade therapy against the programmed cell death 1 (PD-1/PD-L1) axis has emerged with considerable benefits in the immunotherapy of various cancers. The use of MSNs for checkpoint blockade has been reported to improve its efficacy. For example, Li et al. constructed the metal-organic-framework (MOF)-gated MSNs assembled with immunology-associated molecules, including the antigens, soluble immunopotentiators, etc. [418]. The metal-organic-framework gatekeeper protects these loaded molecules from off-target release. Combining these MOF-gated MSN-loaded immunology-associated molecules with systemic PD-1 blockade therapy showed a remarkable synergistic anti-cancer effect with enhanced anti-tumor immunity. The required doses of the anti-PD-1 antibody in this combined immunotherapy was 1/10 of what was used for PD-1 blockade monotherapy in lymphoma tumor-bearing mice. These data showed that MOF-gated MSNs are capable of the efficient delivery of immunology-associated molecules and improving the treatment efficacy of PD-1 blockade therapy. Since inhibition of the glycogen synthase kinase 3 has also been shown to suppress PD-1 expression and interfere with the PD-1/PD-L1 axis, Allen et al. constructed the lipid bilayer-coated MSNs loaded with the GSK3 inhibitor AZD1080 for the immunotherapy of colorectal, pancreatic, and lung cancers [419]. Intravenous administration of these lipid bilayer-coated MSNs led to improved delivery of AZD1080, tumor shrinkage, accompanied by the eradication of tumor cells by CD8+ leukocytes, and reduced PD-1 expression. This study supports the feasibility of MSN-based carriers for the delivery of GSK3 inhibitors in anti-cancer immunotherapy. Besides, Choi et al. formulated an FDA-approved iron oxide nanoparticles ferumoxytol (Fer)-capped immune checkpoint blockade (PD-L1 antibody)-loaded ultra-large pore MSNs (ICB-UPMSNPs) for a sequential MRI-guided local immunotherapy in prostate cancer after cabazitaxel chemotherapy [420]. The highly porous framework of ICB-UPMSNPs allowed the high loading of PD-L1 antibodies. The Fer cap of the MSN pores prolonged the duration of PD-L1 antibody release and provided the MRI visibility of ICB-UPMSNPs. Cabazitaxel chemotherapy elevated PD-L1 expression and induced immunogenic cell death. The sequential MR image-guided local delivery of Fer-ICB-UPMSNPs led to significant inhibition of tumor growth and tissue-specific adoptive immune response in the TRAMP C1 prostate adenocarcinoma cell-transplanted mouse model, pretreated with Cbz chemotherapy. These findings indicated that MRI-guided local immune checkpoint blockade immunotherapy using Fer-ICB-UPMSNPs provides an additional therapeutic strategy for patients after standard chemotherapy treatment. Furthermore, Xie et al. combined an MSN-based strategy and anti-PD-1 immunotherapy to treat melanoma [421]. MSNs were loaded with glucose oxidase (GOx) and then functionalized with cancer cell membranes, thereby generating the CMSN-GOx to escape immune clearance. CMSN-GOx was able to ablate tumors and promoted the maturation of dendritic cells to elicit the anti-tumor immune response. Furthermore, the combination of CMSN-GOx and PD-1 inhibition showed a synergistic anti-tumor effect in vivo. Combining the CMSN-GOx starvation therapy and anti-PD-1 immunotherapy may open new horizons for clinical applications of MSN-based systems in anti-cancer therapy.

To further enhance anti-tumor effects, MSNs have also been explored as a multifunctional system for the combination of immunotherapy with PDT in various cancers, including colon [422,423,424], breast [425], and lung cancers [426]. For example, Xu et al. synthesized biodegradable MSNs (bMSNs) for theragnostic PET-guided PDT and neoantigen-based cancer vaccination. These bMSNs were co-loaded with multiple neoantigen peptides, CpG oligodeoxynucleotide immune adjuvants, and PS. PET imaging revealed that, after the intravenous administration, bMSNs tended to accumulate at the tumor sites demonstrating the efficient delivery of MSNs. Moreover, laser irradiation-induced PDT could also recruit dendritic cells to the tumor sites and elicit the neoantigen-specific and tumor-infiltrating cytotoxic T lymphocyte responses. In vivo studies indicated strong anti-tumor efficacy of this MSN-based PET-guided PDT and neoantigen-based cancer vaccination against local and distal tumors [146]. Similarly, Im et al. constructed a type of hypoxia-responsive MSNs to improve the intracellular uptake of nanocarriers and the delivery of adjuvants to dendritic cells, thereby improving the efficacy of immunotherapy using PDT [427]. The PDT further promoted the generation of immunogenic debris and the recruitment of dendritic cells to the tumor sites, followed by elevated antigen presentation. In another study, Wang et al. also reported similar synergistic anti-metastatic effect by the combination of immunotherapy, MSN-assisted PDT, and magnetic hyperthermia in primary and metastatic breast cancers [428]. Another example of the combination therapy, employing both immunotherapy and photothermal therapy, was the study by Seth et al., which demonstrated the potential of multifunctional NIR-responsive core-shell MSNs, in photothermal ablation of cancer cells and triggering the release of immunomodulatory drugs to initiate a series of immunological events. Taken together, these multifunctional NIR-responsive core-shell MSNs may offer great synergistic potential by the combination of immunotherapy and photothermal therapy [429].

In addition to PDT, MSNs have also been used for the combination of immunotherapy and photothermal therapy. Qian et al. loaded MSNs with carbon nanodots (CD@MSNs) that can gather dispersive carbon nanodots with enhanced photothermal effect and improved targeting accumulation, thus achieving photothermal imaging-guided photothermal therapy [430]. Notably, CD@MSN-mediated photothermal therapy can stimulate the proliferation and activation of natural killer cells, macrophages, and cytokine secretion, leading to the immune-mediated inhibition of tumor metastasis. In another study, dendritic large-pore MSNs (DLMSNs) were used for photothermal tumor ablation and immune remodeling with remarkable therapeutic effects on triple-negative breast cancer [431]. Homogenous cancer cell membranes were coated on these DLMSNs, and the anti-PD-1 peptide was then conjugated through a polyethylene glycol (PEG) linker. The ordered large-pore structure of MSNs allowed the deposition of both the immune adjuvant R848 and copper sulfide nanoparticles with high photothermal conversion efficiency within MSNs. In response to the photothermal effect, R848 was released to produce vaccine-like functions, and anti-PD-1 peptide was detached to boost the anti-tumor effect of T lymphocytes in the tumor microenvironment. Altogether, these findings developed an MSN-based multifunctional nanoplatforms to enhance the treatment outcome in triple-negative breast cancer.

Overall, the application of MSNs provides a comprehensive approach to eradicate cancer cells by designing and tuning properties that are not possible with other therapeutic drugs. With MSNs evolving in various dimensions, forms, functionalities, and properties, these stimuli-sensitive vehicles shed light on a promising generation of cancer therapeutics with improved targeting ability, safety, and efficacy.

## 5. Biocompatibility and Safety of MSNs

The safety and biocompatibility assessments of nanomaterials are indispensable concerns prior to any in vivo application. In general, amorphous silica particles are degraded over time into non-toxic ortho-silicic acid (Si(OH)_4_) [25]. Silicic acid is water-soluble, which can contribute to bone mineralization and is also excreted via urine [432,433]. MSNs can be completely degraded over a month in simulated body fluid [25,434,435,436], in the cells [320], and in the body [80,437]. However, silica degradation is relatively difficult under physiological conditions. This is why FDA considered silica as “Generally Recognized As Safe (GRAS)” [20,21,22,23]. Several groups have conducted research to evaluate the genotoxicity, cytotoxicity as well as blood and tissue compatibility of MSNs. The results significantly vary based on different factors such as the size, shape, concentration of MSNs, the treatment period, and the studied cell type [438,439]. For example, one study reported negligible genotoxic effect in HT-29 [440], whereas another study found a significant effect in human embryonic kidney cells after overnight exposure to MSNs [439]. Interestingly, microarray analysis showed that overnight exposure to MSNs significantly increased 579 genes and decreased 1263 genes in human embryonic kidney 293 cells [441].

In cytotoxicity assays, MSNs are generally regarded safe in normal and cancer cell lines (e.g., HeLa, MCF-41, A549, etc.) [144,442]. However, particle-dependent effects such as size and morphology [443,444] play critical roles in determining the cytotoxicity of MSNs. For example, rod-shaped MSNs appeared to be more cytotoxic than spherical MSNs with increasing aspect ratios in Hella cells [445]. Despite the importance of the aforementioned parameters, choosing a proper in vitro cell viability assay for the cytotoxicity analysis is crucial for setting an appropriate dosage. This improves the optimal therapeutic effect while minimizing the potential side effects [120]. Braun et al. performed different viability assays to examine the cytotoxic effect induced by four different functionalized MSNs in the cervical carcinoma TZM-b1 cell line. The results indicated that the MTT assay apparently overestimates the cytotoxicity of MSNs, while the WST-1 assay showed an opposite effect [446].

If the drug-loaded MSNs are administered by the intravenous (IV) injection, their thrombogenicity, hemolytic activity, and the adsorption of blood proteins on the surface of MSNs, have crucial impacts on the blood biocompatibility of MSNs. It is noteworthy that surface modifications of MSNs with organic moieties, in particular, by PEG, could significantly improve their hemocompatibility [6,447,448]. The surface modifications of MSNs with PEG can decrease the interaction between positively charged trimethylammonium groups of the lipid bilayer of red blood cells (RBCs) and negatively charged silica [448,449]. The other concern is the half-life and the colloidal stability of MSNs after the conjugation with functional groups. MSNs, with a size of less than 50 nm, exhibit adequate passivation with hydrophilic molecules, which leads to long-term blood circulation [450,451]. However, the addition of, for example, imaging agents can increase the MSNs size to around 100–400 nm and thus have a negative impact on their blood half-life. Nonetheless, the information regarding the half-life of MSNs in the bloodstream is very limited and demands more in-depth in vivo investigations [436,452,453].

Furthermore, the in vivo tissue compatibility of MSNs indicated that repeated dosages of lower than 80 mg kg^−1^ could be well-tolerated by mice, whereas a very high dosage of 1.2 g kg^−1^ was toxic, demonstrated by marked pathological changes [15,454]. These findings were further confirmed by in vivo studies using dosages of 100–200 mg kg^−1^ MSNs functionalized with imaging agents in hMSCs [328,329]. However, the long-term biocompatibility of MSNs has not been thoroughly investigated, given the fact that this research area is in its infancy [455]. Nonetheless, a recent report [456] on the long-term biocompatibility of PEGylated HMSNs in mice indicated that the intravenous injection of high dosages of these nanocarriers (20 mg kg^−1^) did not show any toxic side effect on the analyzed tissues including, the liver, heart, spleen, kidney, and lung for a period of 30 days. Despite the numerous reports on MSNs, none of the MSN-based formulations have received FDA approval for clinical applications. This is especially true when considering the complexity of different types of MSNs, such as physical properties, surface chemistry, and functionalization. To achieve clinical translation, fundamental issues such as biodistribution, toxicity, and the final destination of MSNs in the body need to be addressed [144,457]. In addition, a simpler formulation of MSNs may be a helpful strategy to clinically validate the satisfactory biocompatibility result shown in animal models [110].

## 6. Conclusions

**Figure 10:**
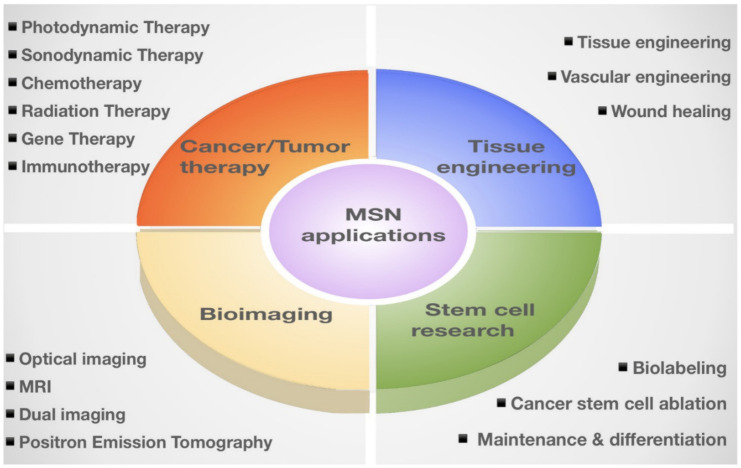
**The application of MSNs in medicine.** MSNs show great potential for various biomedical applications, including anti-cancer/tumor therapy, bioimaging, tissue engineering, and stem cell research.

In this review, we focused on the latest progress in the diagnostic and therapeutic applications of MSNs. As stated in the multitude of reports, MSN-based delivery systems show outstanding potentials for various medical applications, including cancer therapy, tissue engineering, and bio-imaging (Figure 10). However, utilizing these smart nano-carriers in the clinic and their commercialization have yet to be investigated. Their unique physicochemical properties, such as tunable size and morphology, high surface area, and versatile surface modifications, allow the generation of multifunctional MSNs capable of delivering various cargos in a controlled manner. For example, MSN-based imaging agents offer the possibility of simultaneous diagnostic and drug delivery. However, the introduction of chemical moieties onto the surface of MSNs can cause toxicity and undesirable side effects [458]. In addition, due to the limited active sites on the surface of MSNs, the conjugation of multiple functional groups with proper concentration pose a challenge [112]. It is important to note that when MSNs with single storage space carry different types of drugs, the release of each drug cannot be controlled individually, emphasizing the importance of multi-compartment MSNs [441]. Nevertheless, it is essential to thoroughly understand the behavior of MSNs in the human system to improve their properties for the treatment of human diseases. Meanwhile, the pharmacokinetics and biodegradability of MSNs are prerequisite considerations for future applications. Overall, engineering novel MSNs suitable for clinical applications demands multidisciplinary research on factors involved in every step from preparation and synthesis to the immune system response and efficacy issues. On account of the excellent biocompatibility and functionalities of MSNs, we believe this rapidly growing nanocarrier will revolutionize the smart biomaterial and facilitate personalized treatments and diagnostic strategies.

## Figures and Tables

**Table 1:** **Update of MSN applications in nanomedicines.** We conducted a literature review and summarized the latest publications regarding MSN applications in tissue engineering, bioimaging, stem cell research, and anti-cancer/tumor therapy.

Section	Updated MSN Applications in Nanomedicines	Page
**4.1**	**Tissue engineering**	12
4.1.1.	—bone tissue engineering	12
4.1.2.	—vascular tissue engineering	14
4.1.3.	—wound healing and antibacterial effects	15
**4.2**	**Bioimaging**	17
4.2.1.	—optical imaging	18
4.2.2.	—magnetic resonance imaging and positron emission tomography	19
4.2.3.	—multi-modal imaging	20
**4.3**	**Stem cell research**	22
4.3.1.	—stem cell maintenance and differentiation	22
4.3.2.	—cancer stem cell ablation	24
4.3.3.	—stem cell labeling	27
**4.4**	**Anti-cancer/tumor therapy**	27
4.4.1.	—photodynamic and sonodynamic therapies	28
4.4.2.	—chemotherapy	32
4.4.3.	—radiation therapy	33
4.4.4.	—gene therapy	35
4.4.5.	—immunotherapy	35

**Table 2:** Classification of material-based traditional MSNs and modern hollow MSNs.

Classifications of MSNs			
Traditional MSNs			Reference
Mobil Crystalline Material (MCM)	Mobil Oil Corporation	MCM-41, MCM-49, MCM-50	[74,75,76,77,78,79]
Santa Barbara Amorphous (SBA)	University of California at Santa Barbara	SBA-12, SBA-15, SBA-16	[69,70,80,81]
Korea Institute of Technology (KIT)	Korea Advanced Institute of Science and Technology	KIT-5, KIT-6	[82,83]
Centrum voor Oppervlaktechemie en Katalyse (COK)	Centre for Research Chemistry and Catalysis	COK-12	[84]
**Modern MSNs**			
Hollow MSNs	-	HMSNs	[73,85,86,87,88,89,90]
